# Binding of Gemini Bisbenzimidazole Drugs with Human Telomeric G-Quadruplex Dimers: Effect of the Spacer in the Design of Potent Telomerase Inhibitors

**DOI:** 10.1371/journal.pone.0039467

**Published:** 2012-06-21

**Authors:** Ananya Paul, Akash K. Jain, Santosh K. Misra, Basudeb Maji, K. Muniyappa, Santanu Bhattacharya

**Affiliations:** 1 Department of Organic Chemistry, Indian Institute of Science, Bangalore, India; 2 Department of Biochemistry, Indian Institute of Science, Bangalore, India; 3 Chemical Biology Unit, Jawaharlal Nehru Centre for Advanced Scientific Research, Bangalore, India; Wayne State University School of Medicine, United States of America

## Abstract

The study of anticancer agents that act via stabilization of telomeric G-quadruplex DNA (G4DNA) is important because such agents often inhibit telomerase activity. Several types of G4DNA binding ligands are known. In these studies, the target structures often involve a single G4 DNA unit formed by short DNA telomeric sequences. However, the 3′-terminal single-stranded human telomeric DNA can form higher-order structures by clustering consecutive quadruplex units (dimers or n-mers). Herein, we present new synthetic gemini (twin) bisbenzimidazole ligands, in which the oligo-oxyethylene spacers join the two bisbenzimidazole units for the recognition of both monomeric and dimeric G4DNA, derived from d(T2AG3)4 and d(T2AG3)8 human telomeric DNA, respectively. The spacer between the two bisbenzimidazoles in the geminis plays a critical role in the G4DNA stability. We report here (i) synthesis of new effective gemini anticancer agents that are selectively more toxic towards the cancer cells than the corresponding normal cells; (ii) formation and characterization of G4DNA dimers in solution as well as computational construction of the dimeric G4DNA structures. The gemini ligands direct the folding of the single-stranded DNA into an unusually stable parallel-stranded G4DNA when it was formed in presence of the ligands in KCl solution and the gemini ligands show spacer length dependent potent telomerase inhibition properties.

## Introduction

The 3′-end of the telomeric DNA plays a crucial role in chromosome stability and in the protection from degradation, fusion or recombination [1]–[3]. Few hundred nucleotides at the terminal end of chromosomes, called telomere, remain single-stranded and are folded into four stranded structures, called the G-quadruplex DNA (G4DNA), a structure induced by Na^+^ or K^+^ ions or by some small organic ligands [4]–[8]. This structure is refractory to telomerase, a ribonucleoprotein of about 170 kDa, which is up-regulated in about 85% of human tumors and is undetectable in most of the normal somatic cells [4], [5].

**Figure 1:**
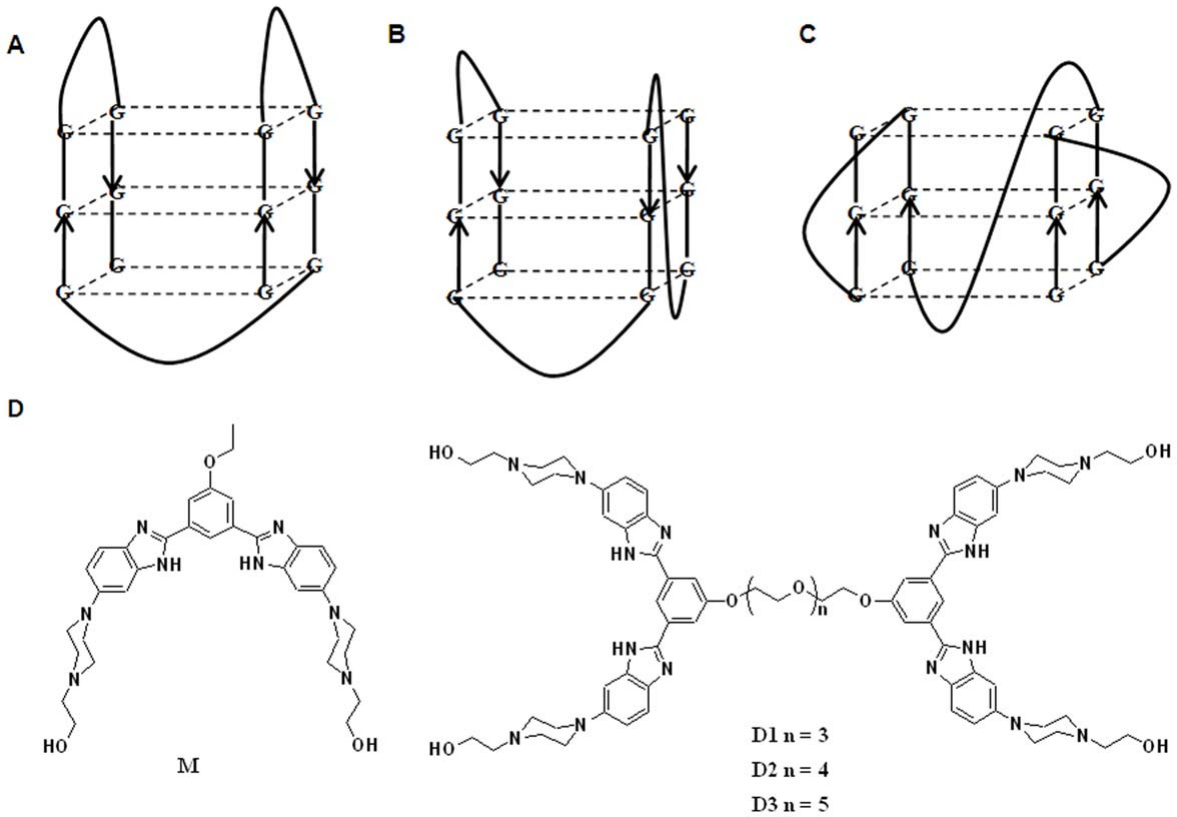
Topology of the G4DNA and the ligands used in the study. (A-C) Folding topology of the monomeric intramolecular G4DNAs as elucidated from NMR or X-ray crystallography: A) basket-type intramolecular G4DNA in Na^+^ solution (NMR) B) hybrid-1 intramolecular G4DNA in K^+^ solution (NMR) and C) propeller-type parallel-stranded intramolecular G4DNA in presence of K^+^ in the crystalline state (X-ray crystal structure). (D) Chemical structures of the ligands used in the present study.

Human telomeric DNA comprises the hexanucleotide 5′-TTAGGG- repeats and these repeats spontaneously fold into two distinct but related hybrid-types, i.e., hybrid-1 (**Figure 1**) or hybrid-2 telomeric G-quadruplexes, depending on the flanking sequences [4], [5], [9]–[11]. DNA sequence, *e.g.*, d(T_2_AG_3_)_4_ and its variants, *e.g.*, d[AG_3_(TTAG_3_)_3_], d[G_3_(T_2_AG_3_)_3_], d[T_2_A(G_3_T_2_A)_3_G_3_T_2_] etc. form a single G-quadruplex loop or a monomer of the G4DNA [9], [11]–[16]. Recent studies show that a 48-mer sequence d(T_2_AG_3_)_8_ and its 50-mer variant sequence d(T_2_AG_3_)_8_TT form dimeric G4DNA structures containing two-folded G-quadruplex units [17]–[19]. It has been proposed that the multiple repeats of this sequence, *i.e.*, d(T_2_AG_3_)_n_ fold and stack, end-to-end, to form compact-stacking structures containing several G-quadruplex units [10], [20], [21]. RNA also forms G-quadruplex structure. Recently, long RNA transcripts of human telomeric repeats (TERRA), *i.e.*, r(UUAG_3_)_n_ have been predicted to form multimeric G-quadruplexes, and the eight repeat sequence, *i.e.*, r(UUAG_3_)_8_ forms a dimeric G-quadruplex [22].

Small molecule ligands that promote and stabilize G4DNA structures are used as potential anticancer agents [4], [5], [23]–[25]. G-quadruplex ligands developed so far have been confined for the recognition of a single unit of G4DNA. Ligands in which two or more G4DNA binding pharmacophore units are linked through a suitable spacer may bind with two or more contiguous G-quadruplex units. Some dimeric (gemini) G4DNA binding ligands have been synthesized recently, but they were investigated with the monomeric G4DNA [26]–[28]. To the best of our knowledge, investigation related to the binding properties of a gemini ligand with a dimeric G4DNA has never been reported.

Using a series of ligands based on 1, 3-phenylene-bis (piperazinyl benzimidazole) previously we have shown the stability, topological changes and induction (in absence of any added cations) of G4DNA derived from the sequence d(T_2_G_4_)_4_, telomeric repeat from *tetrahymena thermophilia* [29] and human telomeric repeat d[(G_3_T_2_A)_3_G_3_] [30]. Herein we describe the synthesis of new gemini ligands in which appropriate bisbenzimidazole monomers are linked via different oligoxyethylene spacers (for synthesis and characterization of the ligands, please see the **Supporting Information**, **Text S1**), and demonstrate their interaction with G4DNAs formed by human telomeric repeats, d(T_2_AG_3_)_4_ and d(T_2_AG_3_)_8_. We also show the ability of these ligands to induce the formation of parallel G4DNA in K^+^ solution. Furthermore, the gemini ligands inhibit the telomerase activity and also display selective cytotoxicity towards the cancer cells.

## Results

Because of their significant cellular membrane permeability, benzimidazoles are used in cytometry, in staining vertebrate chromosomes, radioprotection, enzymatic inhibition and in the stabilization of duplex, triplex and G4DNA [29]–[37]. Herein we employed a monomeric ligand (**M**) [29] having central symmetrical ‘V’-shaped planar core and three of its gemini analogues (**D1**, **D2** and **D3**) in which two monomeric units are joined via oligooxyethylene spacers of different lengths (**Figure 1D**). The angular geometry of the ligand plays an important role in the G4DNA stabilization [29], [36], [38], [39]. Owing to their unusual hydrophilic, lipophilic and biological properties of polyethylene glycol (PEG), incorporation of oligo(ethylene glycol)s as spacer further increases the usefulness of such pharmacophores [40]. Na^+^ and K^+^ ions induce the G4DNA structures and also provide sufficient stability to the G4DNA without any added ligand. Li^+^ ions on the other hand, induce the G4DNA formation, but they do little toward the G-quadruplex stabilization [29], [41]. The structure of the G4DNA formed by four repeat sequence d(T_2_AG_3_)_4_ has been well characterized in various solution conditions [42], [43]. In Na^+^ solution, it forms an intramolecular, anti-parallel basket type G4DNA containing two lateral loops and one diagonal loop, similar to the one reported for d[AG_3_(T_2_AG_3_)_3_] (**Figure 1A**) [42], [44]. In K^+^ solution, it forms mainly a unimolecular structure, having anti-parallel/parallel strands with one propeller and two lateral loops (3+1 hybrid) along with a small percentage of other structures [42]–[44]. For the sequence d(T_2_AG_3_)_8_, a dimeric hybrid-12 quadruplex structure (formed by a hybrid-1 quadruplex at the 5′-end and a hybrid-2 quadruplex at the 3′-end) and a propeller G4DNA have been suggested to be the most stable structure in K^+^ stabilized conditions based on computational studies [19]. This sequence is predicted to form a dimeric structure in Na^+^ solution on the basis of electrophoresis studies [18], but no other studies (including computational) for the system in Na^+^ solution have been reported.

Four buffer systems were used in the present study. One of them has (Na^+^ + LiCl) (10 mM sodium cacodylate having 100 mM LiCl and 0.1 mM EDTA, pH 7.4) and the other three have either of LiCl or NaCl or KCl (100 mM of either LiCl or NaCl or KCl, 0.1 mM EDTA, and 10 mM Tris-HCl buffer, pH 7.4) respectively. Thermal annealing conditions were used to form the G4DNAs from the oligodeoxynucleotides (ODNs) d(T_2_AG_3_)_4_ and d(T_2_AG_3_)_8_ (abbreviated as Hum_24_ and Hum_48_ respectively, see Materials and Methods for further details).

### Circular Dichroism Spectral Titrations

**Figure 2:**
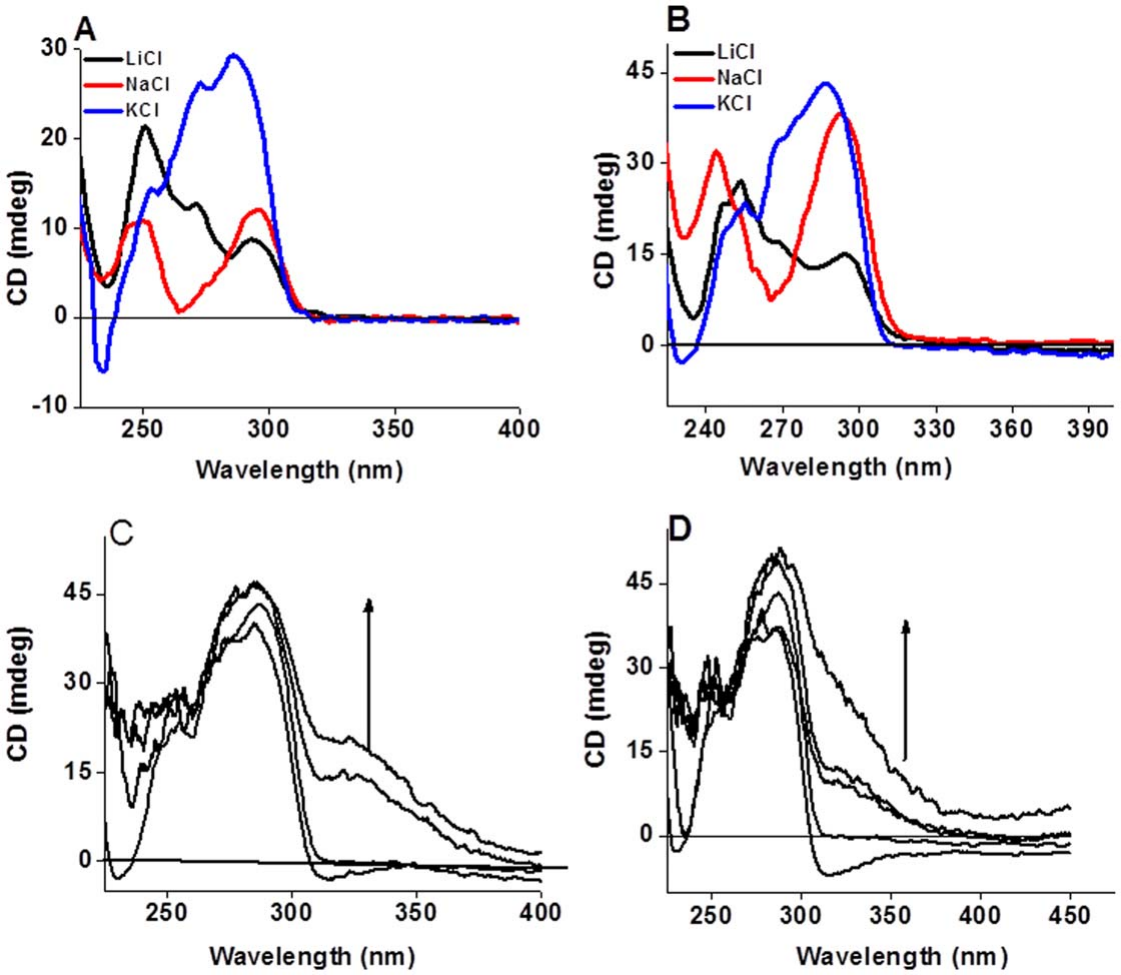
CD spectra of the G4DNA without ligands and CD titrations in KCl solution. Circular dichroic (CD) spectral profiles of G4DNA formed using the Hum_24_ (Panel A) and the Hum_48_ (Panel B), 4 µM DNA in each case, in presence of the indicated monovalent cations (100 mM). (Panels C and D) CD spectral titrations of the pre-formed G4DNA (4 µM strand concn.) formed using the Hum_48_ in KCl buffer (10 mM Tris, pH 7.4 having 100 mM KCl) in presence of **M** (Panel C) and **D3** (Panel D) at ligand: DNA ratio (r) = 10, 20, 30 (for **M**) and r = 5, 10, 15, 20 (for **D3**). Arrows indicate the direction of increments observed in the CD intensity.

Circular dichroism (CD) spectra of the G4DNA formed by Hum_24_ in NaCl and KCl were similar to the ones reported earlier (**Figure 2A**) [9]–[11], [42], [43]. In Na^+^ solution it showed two positive peaks of approximately equal intensity (at 295 and 247 nm) with two valleys (at 265 and 240 nm). Incidentally, both of these spectra had a valley near 265 nm, and not a negative peak as reported [43]. In Li^+^ solution (100 mM Li^+^ and 10 mM Na^+^) it showed two positive peaks (a small peak at 295 nm and large peak at 247 nm) with a hump near 270 nm and two valleys (near 280 and 240 nm). CD spectrum of d(T_2_AG_3_)_4_ recorded in K^+^ solution showed a positive peak near 285 nm, shoulders near 268 and 250 nm, and a negative peak around 240 nm. Such a spectrum is consistent with the ones reported for similar sequences in K^+^ solution [42], [43]. The spectra appeared similar to those observed for other variants, *e.g*., d[A_3_G_3_(T_2_AG_3_)_3_A_2_] and d[(T_2_AG_3_)_3_A_2_] in K^+^ solution but were different from that observed for d[A_3_(T_2_AG_3_)_3_] [9], [11]–[15]. CD spectra for the G4DNA formed with ODN Hum_48_ in all the salt solutions (Na^+^, K^+^ or Li^+^) were again similar to those observed with the Hum_24_ G4DNA, but the intensity of the peaks were higher (**Figure 2B**). Spectra in K^+^ solution are again consistent with the reported ones [13], [18], [43].

Next we prepared the G4DNA from Hum_24_ and Hum_48_ sequences in 100 mM LiCl solution (10 mM Tris.HCl, pH 7.4 having 100 mM LiCl and 0.1 mM EDTA) [44]. CD spectra of G4DNA recorded in Li^+^ solution showed a hump near 277 nm and a peak near 257 nm **(Figure S1)**. This CD spectral profile is different from that recorded in (Na^+^ + Li^+^) solution, indicating that topology of G4DNA is different in both the cases.

We also performed the CD titrations of the pre-formed Hum_24_ G4DNA with gemini ligand **D3** in (Na^+^ + LiCl) buffer (10 mM sodium cacodylate having 100 mM LiCl, pH 7.4) and KCl buffer. There was no structural change in these conditions (**Figure S2**). But the peak intensities changed and a small induced CD (ICD) signal also appeared near 330 nm showing an interaction of the ligands with the G4DNA tetrads and its grooves. Monomeric ligand **M** also furnished similar results (not shown).

The CD titrations of the pre-formed Hum_48_ G4DNA were separately carried out with the monomeric ligand **M** and gemini ligand **D3** in (Na^+^ + LiCl) buffer (10 mM sodium cacodylate having 100 mM LiCl and 0.1 mM EDTA). Interestingly, the intensity of the 295 nm peak increased in both the cases with little effect on the 247 nm peak. In the case of Hum_48_-**M** complexation, three peaks of approximately equal intensity (245, 270 and 295 nm) were observed due to appropriate ligand-DNA interactions (**Figure S3**). But in the case of Hum_48_-**D3** complex, the intensity of the 290 nm peak was much higher and the spectrum was more like that of a K^+^-ion stabilized Hum_48_ G4DNA (**Figure S3**). A significant ICD signal near 327 nm also appeared in both the cases. It is evident that **D3** has a stronger effect than **M** on the Hum_48_ G4DNA. The ICD signal most likely appears due to an interaction of the achiral ligand with the chiral grooves of the G4DNA. The side chains of the ligands should also have their role in the G4DNA groove binding [29].

CD titrations of the pre-formed G4DNA formed in 100 mM LiCl solution (10 mM Tris-HCl, pH 7.4 having 100 mM LiCl and 0.1 mM EDTA) with the ligands **M** and **D3** did not show any topology change. However, a strong ICD signal appeared near 325 nm upon ligand-G4DNA interaction **(Figure S1)**. The interactions of ligands with DNA were quite strong in this case and the CD spectra became distorted after interaction with 12 equiv. of **M**.

The solution structure of K^+^-stabilized G4DNA has significant biological relevance [42], [43]. CD titrations of the pre-formed Hum_48_ G4DNA in K^+^ solution with either **M** or **D3** showed that the 285 nm peak became stronger in both the cases and the negative peak at 240 nm lifted upwards progressively (**Figure 2C, 2D**). An ICD signal near 328 nm also appeared in both the cases. No topology change was apparent in the pre-formed G4DNA after interacting with either of the ligands. Similarly, there was no change in the topology of the pre-formed Hum_48_ G-quadruplex DNA in Na^+^ solution upon interaction with the ligands.

**Figure 3:**
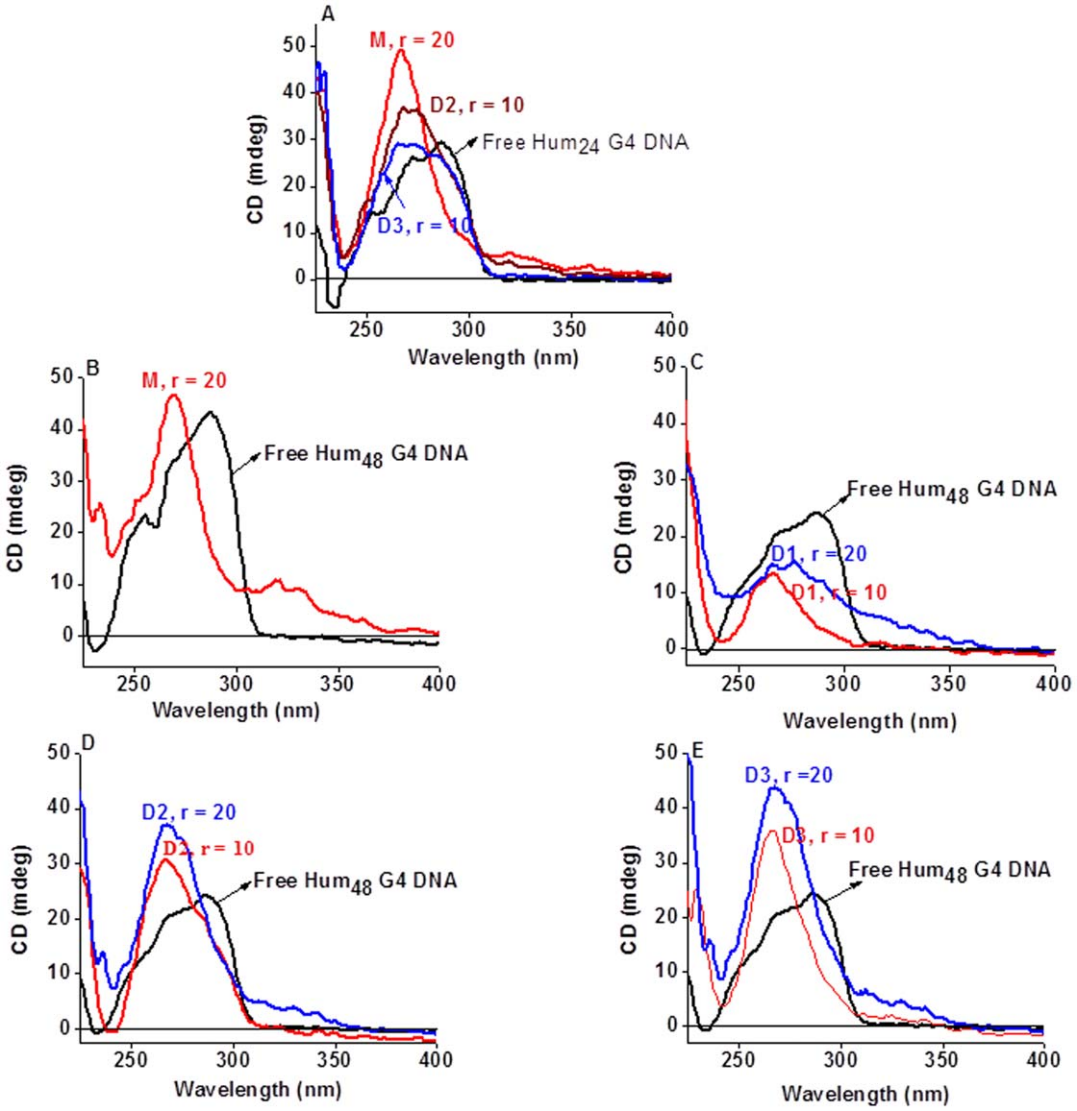
Evidence of change in Topology of the G4DNA using CD spectroscopy. (Panel A) CD spectral profiles of free G4DNA derived from the Hum_24_ DNA (4 µM, black) and G4DNA formed in presence of the indicated ligand in KCl solution. (Panels B to E) CD spectral profiles of free G4DNA derived from the Hum_48_ DNA (black, 4 µM in the case of ‘B’ while 2 µM in other cases) and G4DNA formed in presence of the indicated ligand. Solution (10 mM Tris, pH 7.4 having 100 mM KCl), with or without the ligands, was first heated to 100°C for 5 min and then cooled slowly to room temperature to form the G4DNA in each case.

But significant structural changes occurred in the CD spectral profile of Hum_24_ G4DNA formed (first heated to 95°C for 5 min and then cooled slowly to room temp.) in presence of either the monomeric or the gemini ligands in 100 mM K^+^ solution. Interestingly, at [ligand]:[DNA] ratio (‘r’) of ∼10, all the three peaks merged to give a peak near 265 to 270 nm and a shoulder near 295 nm (**Figure 3A**). The 270 nm peak is broader in the case of **D3**. This profile is similar to the CD spectral profile of the parallel G4DNAs formed by human and many non-human telomere DNA sequences, although a shallow was there at 237 nm and no negative peak was seen [29], [45]–[48].

Hum_48_ sequence also produced similar CD spectra (as with the Hum_24_ sequence) when the corresponding G4DNA was formed from it in presence of either monomeric or gemini ligands in 100 mM K^+^ solution. Each of the ligands **M**, **D2** and **D3** gave rise to a strong sharp peak near 265 nm, while **D1** furnished a broad peak near 275 nm (**Figure 3B to 3E**). The peaks were sharper in case of **M**, **D2** and **D3**. These spectral features are again consistent with that of a parallel G4DNA [29], [45]–[48].

### Thermal Denaturation Studies

In the thermal denaturation experiments, monomer **M** and its gemini counterparts showed characteristic melting curves at 295 nm and evidence of significant stabilization of the pre-formed Hum_24_ and Hum_48_ G4DNAs in 10 mM Na^+^ +100 mM LiCl solution at ‘r’ of 10 (r = 20 in case of **M**) (**Table 1**; **Figure S4**). We have chosen (sodium cacodylate + LiCl solution) because Li^+^ helps in the formation of G4DNA but it has practically no effect on the stability of G4DNA. Then we may have a better idea about the stabilization provided by any G4DNA stabilizing ligand under these conditions [29], [41]. Substitution of electron-donating alkoxy groups on the phenyl ring of benzimidazole is known to increase its DNA binding affinity further [29], [35]. Stabilization was considerably higher in the case of the gemini ligands. Gemini ligand **D3** induced higher stabilization of the Hum_48_ G4DNA than **D2** and the stabilization was even higher when the G4DNA was formed in presence of **D3** (**Table 1**). We also performed the melting experiments in 100 mM LiCl solution (10 mM Tris-HCl, pH 7.4 having 100 mM LiCl and 0.1 mM EDTA) [44]. Ligands provided stabilizations ranging from 6°C to 10°C to the G4DNA **(Table S1)**, indicating the poor interaction with the ligands under these conditions.

**Table 1:** Melting temperatures^a^ of Hum_24_ and Hum_48_ G4DNAs formed in (Na + LiCl)^b^ and NaCl^c^ solutions and G4DNA-ligand complexes ([ligand]:[DNA] ratio ‘r’ = 20 for M and 10 for the gemini ligands respectively).

Entry	ODN/ODN-ligand	LiCl solution		NaCl solution	
		*T*_m_ °C	Δ*T*_m_ °C^d^	*T*_m_ °C	Δ*T*_m_ °C^d^
1.	Hum_24_	37	0	41	–
2.	Hum_24_ + **M**	45	8	47	6
3.	Hum_24_ + **D1^e^**	–	–	–	–
4.	Hum_24_ + **D2**	46	9	49	8
5.	Hum_24_ + **D3**	49	12	52	11
6.	Hum_48_	38	–	42	–
7.	Hum_48_ + **M**	47	9	49	7
8.	Hum_48_ + **D1^e^**	–	–	–	–
9.	Hum_48_ + **D2**	52	14	52	10
10.	Hum_48_ + **D3**	55	17	55	13
11.	Hum_48_ + **D3^f^**	57	19	56	14

a Changes in the circular dichroism spectral peak at 295 nm monitored as a function of temperature using 2 µM strand ODN concentration. **^b^**(Na **+** LiCl solution)  =  (10 mM sodium cacodylate pH 7.4 having 100 mM LiCl and 0.1 mM EDTA). **^c^**NaCl solution  =  (10 mM Tris-HCl, pH 7.4 having 100 mM NaCl and 0.1 mM EDTA). **^d^**Δ*T*_m_ values were obtained from the difference in the melting temperatures of the ligand bound and uncomplexed G4DNA. **^e^**No significant increment in *T*_m_. **^f^**For entry 11, the G4DNA was formed in LiCl buffer (first heated to 95°C for 5 min and then cooled slowly). The results are average of two experiments and are within ±0.5°C of each other.

Next we performed the thermal denaturation experiments in 100 mM NaCl solution. The results were similar to those in the case of (Na^+^ + LiCl) solution (**Table 1**; **Figure S5**). Two gemini ligands provided significantly higher stabilization to G4DNA. Likewise the extent of stabilization provided by each ligand to the pre-formed G4DNA was again similar in KCl solution (**Table 2**; **Figure S6**). Gemini ligands consistently afforded higher stabilization of the G4DNA and the effect of the spacer length was also significant. Thus **D2** with a shorter spacer provided somewhat lower stabilization to the Hum_24_ G4DNA than **D3** which possesses a longer spacer.

**Table 2:** Melting temperatures (*T*_m_)^a^ of Hum_24_ and Hum_48_ G4DNA, ligand bound to pre-formed G4DNA and complexes produced when G4DNA was formed in presence of the indicated ligand at [ligand]:[DNA] ratio ‘r’ = 20 for the monomeric and ‘r’ = 10 for the gemini ligands in 100 mM KCl solution.

Entry	ODN/ODN-ligand	Pre-formed G4DNA		G4DNA formed in presence of the ligand	
		*T*_m_ °C	Δ*T*_m_ °C^b^	*T*_m_ °C	Δ*T*_m_ °C^b^
1.	Hum_24_	46	0	46	–
2.	Hum_24_ + **M**	52	5	55	9
3.	Hum_24_ + **D1**	62	16	70	24
4.	Hum_24_ + **D2**	58	12	79	33
5.	Hum_24_ + **D3**	68	22	84	38
6.	Hum_48_	51	0	51	–
7.	Hum_48_ + **M**	60	9	65	14
8.	Hum_48_ + **D1**	67	16	79	28
9.	Hum_48_ + **D2**	69	18	87	34
10.	Hum_48_+ **D3**	72	21	89	38

a Changes in the circular dichroism spectral peak (at 295 nm for the G4DNA and the pre-formed G4DNA-ligand complex; at 266 nm for the G4DNA-ligand complex formed in presence of ligand) monitored as a function of temperature using 2 µM strand ODN concentration. **^b^**Δ*T*_m_ values were obtained from the difference in the melting temperatures of the ligand bound and uncomplexed G4DNA. For the G4DNA-ligand complexes when G4DNA was formed in presence of the ligand, the solution was first heated to 95°C for 5 min and then cooled slowly. The results are average of two experiments and are within ±0.5°C of each other.

Importantly, the monomer **M** and the gemini ligands provided much higher stabilization to the complex when the G4DNA was formed in the presence of respective compound in 100 mM KCl solution (first heated to 95°C for 5 min and then cooled slowly to room temp.). Gemini ligands formed more stable complexes and the stability of the parallel G4DNA formed in presence of these ligands, increased with the spacer length in both the cases (Hum_24_ and Hum_48_, **Table 2**; **Figure S7**).

During cooling, the Hum_48_ DNA did not show any evidence of any specific structure formation (**Figure S7C**). Cooling of the mixture of Hum_48_ with **D3** however, resulted in a G-quadruplex formation. The cooling curve was not superimposable with the heating curve. Ligand **D3** binds and stabilizes G4DNA driving the ssDNA ↔ G4DNA equilibrium toward G4DNA and therefore this might have accelerated the folding process [49].

### Absorption and Fluorescence Titrations

Gemini ligands showed high G4DNA *vs.* duplex DNA selectivity. Absorption titration (**Figure S8**) results show that the binding affinity of the gemini ligands towards G4DNA increases with the spacer length (**Table S2, Figure S9**). Importantly, the gemini ligand **D3**, with the longest spacer between the pharmacophore units, has the highest affinity for the G4DNA (Hum_48_). **D3** also showed a large difference in the affinity for G4DNA over the duplex DNA.

Gemini ligands having oligooxyethylene spacers are capable of forming non-cyclic crown ether-like conformations in presence of suitable metal ions [28], [50]. Thus the gemini ligands may form ‘hairpin’ type complexes having reduced fluorescence emission and absorbance (**Figure S10**) in K^+^ solution. Interestingly, after complexation with metal ion, their activities in terms of their interaction with G4DNA or duplex DNA do not change [28], [50]. Solutions of gemini ligands (**D1**, **D2** and **D3**) have lower fluorescence emission in 100 mM K^+^ solution. After addition of the pre-formed Hum_48_ G4DNA, the fluorescence intensity started increasing, indicating the binding of ligands with the G4DNA (**Figure S10**). Increment in the fluorescence intensity also depended on the spacer length and **D3** showed the highest affinity with the G4DNA.

#### Telomerase inhibition studies

**Figure 4:**
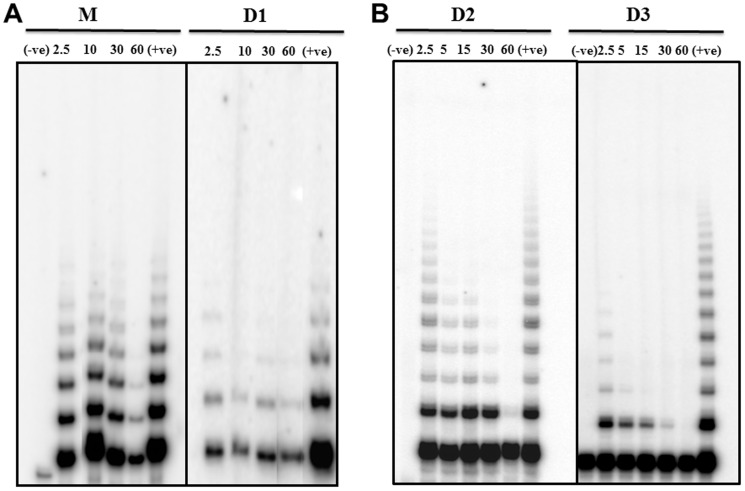
Telomerase inhibitory properties of ligands by TRAP-LIG assay. TRAP-LIG assay was performed using indicated concentrations over each lane with **M** and **D1** (A); **D2** and **D3** (B). A positive control was run with telomerase, but with no ligand. A negative control was run without either telomerase or ligand. Positive and negative control lanes are indicated by + and – labels, respectively. Other lanes contain TRAP reaction mixtures mixed with the indicated concentrations (µM) of each ligand.

The inhibition of telomerase activity is considered as an important indicator for the anticancer activity of a drug. This drug interacts with telomeric G-rich overhang and stabilized G-quadruplex DNA. So this type of molecules acts through dual role as inhibitor of telomere uncapping and telomerase inhibitors [51], [52]. Monomer **M** and gemini ligands **D1-D3** were evaluated for their ability to inhibit human telomerase using conventional two step telomerase repeat amplification protocol (TRAP) assay [23], [38] and modified three step TRAP–LIG protocol assay [53]. On the basis of this assay the ligand which stabilized the G4DNA structure shows telomerase inhibition activity. In modified TRAP–LIG assay the extra step is the removal of the bound and the unbound ligand prior to the PCR step of the assay (see Materials and Methods). Sometimes the ligand has its own inhibitory activity on Taq polymerase and therefore it may affect the PCR amplification. Each ligand was tested at increasing concentrations (in the range of 2.5 µM to 60 µM) against telomerase extract from A549 (human lung carcinoma cell line) cells. As shown (**Figure S11)** by the conventional TRAP assay method, with increasing concentration of ligands a decrease in intensity of the ladders was observed. But in case of TRAP-LIG assay protocol the potency of ligand activity appears to have decreased (**Figure 4** **)**. It requires higher concentration of the ligands to inhibit the same amount of telomerase protein. In TRAP-LIG assay ligand **M** inhibits the telomerase activity at 60 µM (**Figure 4A**). Among the gemini ligands, **D1** inhibits at 30 µM while **D2** and **D3** at <15 µM. The IC_50_ values for the telomerase inhibition by the TRAP-LIG protocol have been also estimated and are shown in **Table 3** and in **Figure S12**. Importantly, the gemini ligand **D3** displays significantly lower IC_50_ values than the other two gemini ligands.

**Table 3:** IC_50_ values against Telomerase found for the different lgands by TTAP-LIG assay^a^.

Entry	Ligand	IC_50_ (µM)	
1.	**M**	55.4	
2.	**D1**	38.6	
3.	**D2**	32.2	
4	**D3**	5.9	

a The results are average of three experiments and are within ±1% of each other.

### Cancer Cell-specific Toxicity

Next we examined the effects of each ligand on three different cancer cell lines, *i.e*., HeLa, A549 and NIH3T3. First we performed short-term cell viability study for 6 h. Eventually a 48 h cytotoxicity assay (MTT assay) was performed with each ligand (**Figure S13**). Each ligand exerted an inhibitory effect and evidently each gemini ligand was more cytotoxic towards the cancer cells than that of the monomeric ligand, **M**, as reflected from the respective IC_50_ values (**Table S3**).

**Figure 5:**
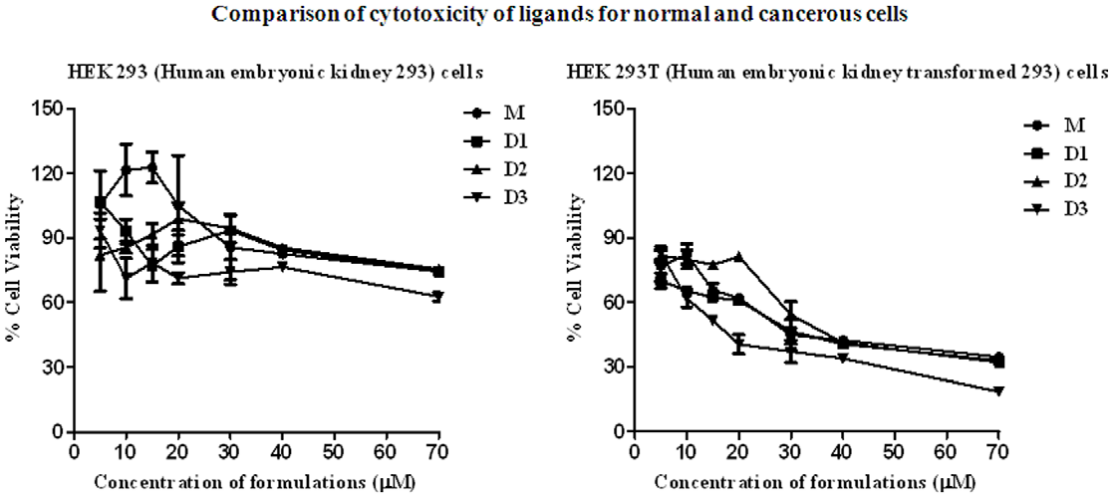
Selective toxicity of ligands toward cancer cells. Effect of the ligands on the cell viability after 48 h exposure of HEK293 (normal) and HEK293T (cancerous) cells with each ligand at different concentrations as measured by MTT (methyl thiazolyl tetrazolium) assay. Each experiment was repeated six times.

To evaluate the long-term effects of each ligand on the growth of cancer cells, sub-cytotoxic concentrations (4 µM) of ligands were employed to lessen acute cytotoxicity and other off-target events. Generally, each ligand exerted significant inhibitory effect with all the three cell lines examined (**Figure S14**). To ascertain the inhibitory effect of ligands towards cancer cells over normal cells, we performed MTT assay on HEK293 normal cells and HEK293T cancer cells. Each ligand was found to be significantly more toxic towards the cancer cells than the normal cells at all the concentrations tested (**Figure 5**). All the ligands are effective at much higher IC_50_ values (48 h treatment) for the HEK293 cells (>150 µM, **Table S3**) than that for the transformed HEK293T cells (<30 µM). Among all the ligands, **D3** showed the lowest IC_50_ value (15 µM) of toxic response toward the HEK293T cancer cells. Further we have tested the cancer cell selectivity study with very well-known G-quadruplex binding molecule H2TMPyP4. This molecule was found to be non-selective for the normal embryo kidney HEK293 cells against cancerous HEK293T cells. Further the IC_50_ values of this compound for each cell line tested were found to be significantly higher than 100 µM (**Figure S15**).

### Molecular Modeling

To gain more insights into the nature of binding of the ligands with the telomeric G4DNA, an approach that combined molecular docking and MD simulations, was performed. Higher order arrangements with several G-quadruplex repeats feasible *in vivo* are allowed as a consequence of the length of the telomeric DNA. However, no high resolution structural data are still available for these longer sequences and such sequences may indeed be difficult to study by the usual NMR or crystallographic methods. The higher order structures and the stability of the full length overhang must be known in order to fully understand the nature of interactions of DNA with the telomerase and other telomere-binding proteins. This structural information on the higher order human telomeric G4DNA formed under physiologically relevant conditions is further necessary for the structure based rational drug design.

Based on a previous report [19] we have used the molecular modeling methods to build a model of G4DNA dimer from the extended human telomeric DNA sequence. Two high resolution NMR structures for the human telomeric sequence in K^+^ solution are available, and these are known as hybrid-1 and hybrid-2 G4DNAs, respectively. These are made up of 26-mer DNA, and were used to build the dimeric G4DNAs [10]. These two structures are highly dominant in a native, non-modified human telomeric DNA sequence in K^+^ solution. Both structures contain three G-tetrads linked mixed parallel/antiparallel G-strands. They differ in their loop arrangements, strand orientation, G-tetrad arrangements, and capping structures. Hybrid-1 structure has sequential side-lateral-lateral loops with the first TTA loop adopting the double chain-reversal conformation. The hybrid-2 structure has lateral-lateral-side loops with the last TTA loop adopting the double chain-reversal conformation. Both hybrid structures contain three parallel G-strands and one anti-parallel G-strand, five *syn* guanine and asymmetric G-arrangement. The first G-tetrad (from the 5′-end) has a reversed G-arrangement from the other two G-tetrads. For the hybrid-1 structure, the first G-tetrad is (*syn*: *syn*: *anti*: *syn*) and the bottom two are (*anti*: *anti*: *syn*: *anti*), whereas for the hybrid-2 structure, the first G-tetrad is (*syn*: *anti*: *syn*: *syn*) and the bottom two are (*anti*: *syn*: *anti*: *anti*) (**Figure S16**). Using these two monomeric motifs as building blocks, we built a dimeric model of G4DNA, named hybrid-12, which was formed by putting the hybrid-1structure at 5′-end and the hybrid-2 structure at the 3′-end. We refined the initial structures using molecular dynamics simulations. After an equilibration period of 250 ps, the model was subjected to an unrestrained molecular dynamics trajectory of 8 ns duration (**Figure S17**).

**Figure 6:**
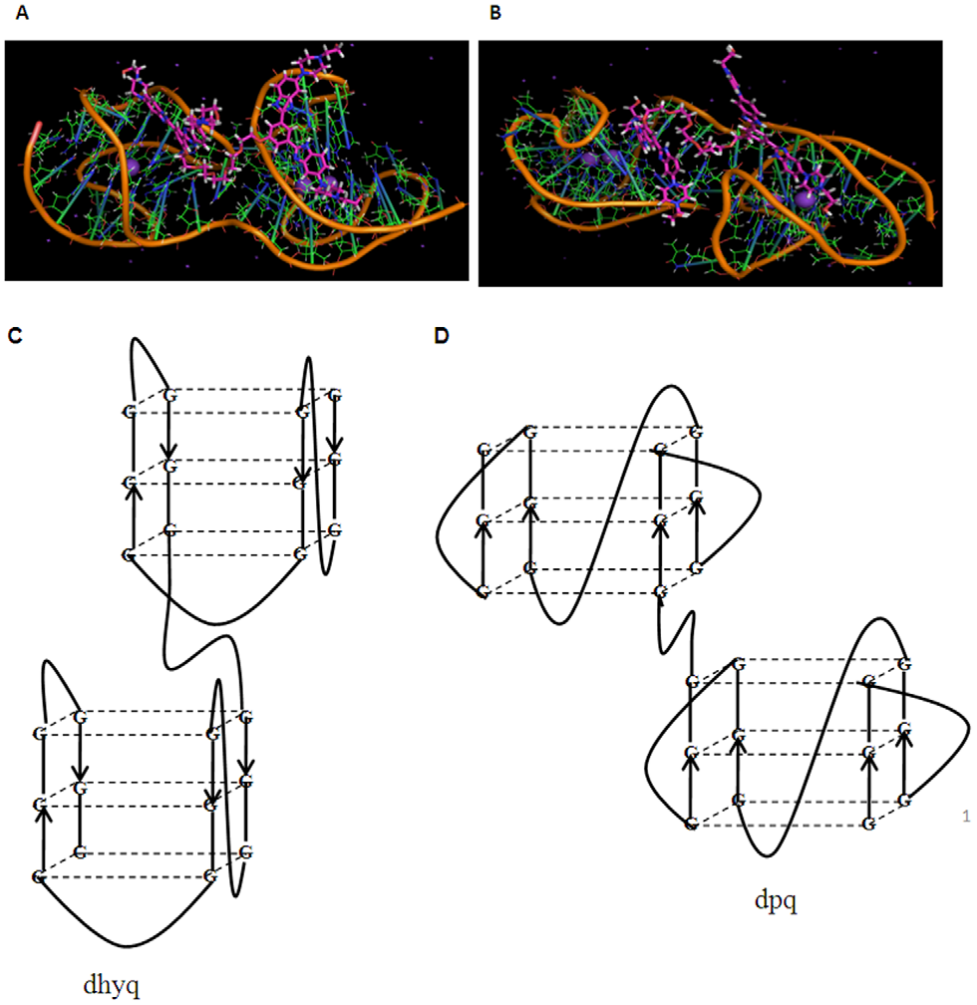
Simulated structures of the dimeric G4DNA-gemini ligand complexes and topology change of the G4DNA. Simulated structures of (A) **D2**-dimeric G4DNA complex and (B) **D3**-dimeric G4DNA complex are shown as a ‘stick’ model. In the case of **D2**, both bisbenzimidazole units bind with the grooves of different G4 DNA units. In the case of **D3**, one bisbenzimidazole stacks on the 5′-surface of the G-quartet plane while the other bisbenzimidazole binds to the groove of the G-quadruplex. K^+^ ions present in the central cavity are shown in cyan colour. Ligand is shown in sticks model colored by atom type (magenta-blue). Hybrid G4DNA is represented as a cartoon form (green-orange). (C and D) Proposed structures of the dimeric G4DNA formed from longer human telomeric repeats: C) hybrid dimeric G4DNA (dhyq) formed in K^+^ solution; D) parallel dimeric G-quadruplex DNA (dpq) formed in K^+^ solution in presence of various ligands (**M** or **D**s).

After generating the stable dimeric G4DNA structure, molecular docking studies were first carried out to predict the possible interaction sites between the dimeric ligands and the G4DNA. We used a 1∶1 stoichiometry for the docking of the dimeric ligands with the dimeric G4DNA by assuming that one bisbenzimidazole unit of a dimeric ligand stack on one G4-plane of the first quadruplex unit of the dimeric G4DNA while the second bisbenzimidazole unit stacks on the G4-plane of the other quadruplex unit of the same dimeric G4DNA. From the docking results it is apparent that due to the steric hindrance on the top of the G-tetrads by the AAA and TT overhangs, the flexible ligands could be readily accommodated in the groove regions of the dimeric hybrid structure. On the basis of the docking results, the lowest final docked energy ligand-G4DNA complexes were chosen and then MD simulations (8 ns) were performed separately on the two complexes formed by dimeric G4DNA with the ligands **D2** and **D3**. All the models were quite stable during the dynamics runs (**Figure 6A**; RMSD values are given in **Figure S18**).

## Discussion

The ligands used in this study possess a planar ‘V’-shaped bisbenzimidazole unit as the core, the dimension of which matches closely with those of the G-tetrads derived from human telomeric repeats. Furthermore, one such bisbenzimidazole unit covers 3 bases of the G-tetrad and is able to provide high stabilization of the G4DNA [29], [38]. As per the CD spectral titrations upon interaction with each ligand, no structural change in the pre-formed Hum_24_ G4DNA in (Na^+^ + Li^+^) solution (10 mM Na^+^ and 100 mM Li^+^, **SI**) was observed. But there were clear changes in the CD spectra of (Na^+^ + Li^+^) stabilized Hum_48_ G4DNA after complexation with the monomeric ligand **M**. The CD spectra of the complex resembled to that of the CD spectra of the K^+^ stabilized d[AG_3_(T_2_AG_3_)_3_] quadruplex [9]–[11], [42], [43]. The changes were more prominent in the case of Hum_48_-**D3** titrations where the CD spectra were similar to that of the K^+^-stabilized Hum_48_ G4DNA (**Figure S3**), indicating stronger interaction between the ligand and G4DNA. This was further corroborated from the observation of a strong ICD signal in each case and from the results of the thermal denaturation experiments.

As K^+^ ion provides higher stabilization to the G4DNA, there was no change in the topology of both pre-formed G4DNAs (Hum_24_ and Hum_48_) after interaction with the monomeric and the gemini ligands. However, the ICD signal observed in all the cases, indicate significant interactions of the ligand with the chiral grooves of the G4DNA after satisfying the G-tetrad stabilization.

‘V’-shaped ligands based on 1, 3-phenylene-bis (piperazinyl benzimidazole) have already been shown to change the topology of hybrid G4DNA derived from the 24-mer *Tetrahymena* sequence [29], [38] Similarly, electrophoretic data of the G4DNA derived from the 21-mer human telomeric repeat d[G_3_(T_2_AG_3_)_3_] complexed either with **M** or **D1** indicate the formation of parallel G4DNA conformation, when it was formed in presence of the ligands [30]. In the present case, the CD titration data reveal that the ligands direct the folding of ‘unstructured’ single-stranded DNA (Hum_24_ and Hum_48_) into a parallel- stranded G4DNA when it is formed in presence of a ligand in 100 mM K^+^ solution. This phenomenon was also observed for the 21-mer DNA d[G_3_(T_2_AG_3_)_3_] in presence of the polyethylene glycol (PEG) in K^+^ solution, where PEG mimics cellular molecular crowding conditions [14]. Telomeric sequence d[AG_3_(T_2_AG_3_)_3_] is known to form parallel G-quadruplex crystals from K^+^ solution [13] and monomeric ligand **M** is able to cover the G-tetrad of this G4DNA [29]. **M** also forms stable, parallel G4DNA with the sequence d(T_2_G_4_)_4_ in absence of any added cation [29]. In other words the pharmacophore unit of **M** has high affinity towards G4DNA. So a high concentration of **M** in the vicinity of DNA in K^+^ solution should be responsible for the parallel folding of DNA. In case of the gemini ligands, the oligooxyethylene spacer also has a role in the G4DNA folding process. There was a sharp intense peak near 266 nm when the Hum_24_ G4DNA was formed in presence of **M**. However, there was a broader peak near 270 nm in presence of either **D2** or **D3**, having the same core unit as **M** (**Figure 3**). **M** binds with one G-tetrad at a given time. On the other hand the gemini ligands bind with two G-tetrads of the same G4DNA (preferably through end-stacking), or with the two G-tetrads from two different G4DNAs [23]. Appearances of broader peaks near 270 nm in presence of D2 or D3-Hum24 complexes possibly originate due to this competition in the binding modes. Again a sharper and more intense peak near 269 nm was observed in the case of Hum_48_-**M** complex when the G4DNA was formed in presence of the ligand in K^+^ solution (**Figure 3**). However, in presence of the gemini ligands, different results were seen. Smaller and broader peaks near 267 nm were seen in case of the Hum_48_-**D1** complex. It could be due to shorter spacer length in **D1** (a tetraethylene glycol spacer, 11 atom-long), which may not provide sufficient length necessary between the two bisbenzimidazole units to stack either on each of the two terminal G-tetrads from the same G4DNA unit or from two different G4DNA units of the same Hum_48_ sequence. Ligand **D2** however, afforded sharper peaks than **D1** because of the presence of a longer spacer (14 atom-long), while **D3**, having 17 atom-long spacer afforded the most intense peaks in their CD spectra (**Figure 3D** **)**. These results indicate that the spacer length of the gemini ligands assumes critical role in the formation of parallel Hum_48_ G4DNA.

**D1** did not provide any significant stabilization to the pre-formed G4DNA (Hum_24_ and Hum_48_) in (Na^+^ + Li^+^) (100 mM Li^+^ +10 mM Na^+^) and 100 mM Na^+^ solutions (**Table** **1**). This may be due to the shorter spacer in **D1**, which forms a pseudocyclic structure in presence of Na^+^ ions [50]. Other two gemini ligands **D2** and **D3** provided 11°C to 17°C stabilization in both the cases showing the role of the spacer (**Table** **1**). Interestingly the stabilization was higher when the G4DNA was formed in presence of **D3** in either case, indicating a stronger binding of the ligand with the G-tetrad. Gemini ligands provided higher stabilization, to Hum_24_ and Hum_48_ G4DNAs in 100 mM K^+^ solution, increasing with their spacer length, than **M**, although **D2** provided a slightly lower stabilization of the Hum_24_ G4DNA (**Table 2**). The effect of the spacer length was more apparent when the G4DNAs (Hum_24_ and Hum_48_) were formed in presence of ligands in 100 mM K^+^ solution.

In the solution containing alkali metal ions, gemini ligands fold into pseudocyclic structures due to the presence of oligooxyethylene spacers and their emission spectra have lower intensities [28], [50]. This pseudocyclic structure could be partly or fully opened in the presence of G4DNA. Hence, the changes in the fluorescence intensity would be even greater when interactions occur with such DNA structures [28]. In other words, DNA binding properties of the gemini ligands complexed with metal ions are retained. The gemini ligands may also be protected efficiently by DNA by the exclusion of solvent or the exclusion of oxygen within the solvent. In our case, gemini ligands possess sufficient, spacer length-dependent affinity (**D3**> **D2**> **D1**), for G4DNA in presence of K^+^ or Na^+^. In case of **D1**, having the shortest spacer, which form pseudocyclic complex with Na^+^ ions, the complex may not be opening because the shorter spacer could not afford the length necessary for the two bisbenzimidazole units of **D1** to bind with different G4-planes of the Hum_24_ G4DNA or the different quadruplex units of the Hum_48_ G4DNA.

The results of the TRAP-LIG assay again demonstrate the role of the spacer length on the inhibition of telomerase activity by the gemini ligands. The ligand **D3** having 17 atom long spacer gave the highest telomerase inhibition (**Figure 4**). Short and long term cell survival assays further indicated the potency of the gemini ligands with general spacer length effect. Moreover, the ligands were found to be selectively detrimental to cancer cells than the corresponding normal cells (**Figure 5**).

Molecular modelling studies suggest that due to the steric crowding of the G-tetrads by overhanging of AAA and TT, both the bisbenzimidazole units of the 14 atom spacer based ligand **D2** accommodate itself in the groove of the G-quadruplex created by the TTA lateral loops (**Figure 6A**), and the spacer adheres to the surface of the TTA loops. On the other hand **D3** with more flexible and longer spacer showed two modes of binding, where one of its bisbenzimidazole scaffold stacks on the 5′-surface of the G-quartet plane while the other bisbenzimidazole binds to the groove of the second G4 DNA unit and the spacer interacts with the surface of the loop (**Figure 6B**).

In summary we have developed four bisbenzimidazole based ligands (one monomeric and three gemini) and compared their propensity toward interaction with certain human telomeric sequences possessing multiple G4DNA units. Though each ligand provided considerable stabilization to G4DNA, gemini ligands exhibited significantly higher affinity towards the G4DNAs, which increased with the length of the oligooxyethylene spacer between the two bisbenzimidazole units. The ligands directed the folding of the telomeric DNA into an unusually stable parallel-stranded structure when G4DNA was formed along with the ligand in K^+^ solution. We propose a plausible model for the parallel-stranded dimeric G4DNA (**Figure 6D**).

Gemini ligands inhibited the telomerase activity *in vitro*, and also exhibited potent and selective cytotoxicity against cancer cell lines as a function of their spacer lengths (**Figure S19**). These ligands might be more useful *in vivo* because of the existence of multimeric G4DNAs in the human genome [54], [55]. In the present study, we further show that gemini ligands have significantly higher selectivity for G4DNA over duplex DNA and the selectivity increases with the increase in the spacer length. Hence the interference caused by their interaction with the duplex DNA *in vivo* would likely to be minimal. In this regard, the dimeric (and/or multimeric) ligands, linked via suitable spacers of appropriate length may serve as more potent anticancer agents by targeting telomeric multimeric G4DNA.

## Materials and Methods

### Materials

All materials and reagents were purchased from the best known commercial sources. All cell lines used in this work were procured from ATCC (USA) as described [56].

### Synthesis

Monomeric compound **M** has been synthesized as reported previously [29]. Dimeric compound (**D3**) has been synthesized as described in the supporting information.

### Oligonucleotides

HPLC purified oligodeoxyribonucleotides (ODN) d(T_2_AG_3_)_4_ and d(T_2_AG_3_)_8_ (abbreviated as Hum_24_ and Hum_48_ respectively) were purchased from Sigma, Genosys, Bangalore. The purity of each ODN was confirmed using high resolution sequencing gel. The concentration of each ODN was determined from the absorbance measurements at 260 nm based on their molar extinction coefficients (*ε*_260_) 244600 and 487600 respectively for d(T_2_AG_3_)_4_ and d(T_2_AG_3_)_8_.

### G4DNA Formation

Single-stranded ODNs Hum_24_ and Hum_48_ were dissolved in required buffer at the indicated concentrations. The solution was first heated to 95°C for 5 min, and then cooled slowly to room temperature over a period of 24 h.

### Circular Dichroism Spectroscopy, Fluorescence Spectroscopy, UV-vis Absorption Titration Experiments and Telomerase Repeat Amplification Protocol (Conventional TRAP Assay)

These studies were performed following the procedure as reported earlier [29], [38].

### *T*_m_ Studies

*T*_m_ measurements were performed on a Jasco J - 810 CD spectropolarimeter in a quartz cell of 10 mm path length equipped with a thermal programmer. CD change was monitored at 295 nm (for G-quadruplex without drugs) and ligand-G4 ligand complex in either (Na **+** LiCl solution) (10 mM sodium cacodylate pH 7.4 buffer having 100 mM LiCl and 0.1 mM EDTA) or NaCl or KCl solution  =  (10 mM Tris-HCl, pH 7.4 buffer having 100 mM NaCl or KCl and 0.1 mM EDTA. CD spectral changes were also observed at 266 nm for the drug/DNA complex when G-quadruplex has formed in presence of ligands at KCl buffer. The temperature was raised from 20 or 25 to 95°C at the rate of 1.0°C/min with a stability of 0.2°C. The transition melting temperature, *T*_m_ was determined from the first derivative of the CD versus temperature plot. The stock solution of each new ligand was prepared in DMSO and diluted in the required buffer prior to use.

### TRAP-LIG Assay [53]

The TRAP assay was performed using a three-step TRAP–LIG procedure: (i) primer elongation by telomerase and addition of ligand, (ii) subsequent removal of the ligand, and (iii) PCR amplification of the products of telomerase elongation.

#### Step 1

This was carried out by preparing a master mix containing 0.1 µg of TS forward primer (59-AAT CCG TCG AGC AGA GTT-39), TRAP buffer (20 mM Tris–HCl, pH 8.3, 68 mM KCl, 1.5 mM MgCl_2_, 1 mM EGTA, and 0.05% [v/v] Tween 20), dNTPs (125 µM each), and protein extract (500 ng/sample) diluted in lysis buffer (10 mM Tris–HCl, pH 7.5, 1 mM MgCl_2_, 1 mM EGTA, 0.5% CHAPS, 10% glycerol, 5 mM β-mercaptoethanol, and 0.1 mM 4-(2-aminoethyl)-benzenesulfonyl fluoride. The PCR master mix was added to tubes containing freshly prepared ligand at various concentrations and to a negative control containing no ligand. The initial elongation step was first out carried at 30°C for 10 min, then at by 94°C for 5 min and finally maintaining the mixture at 20°C.

#### Step 2

To purify the elongated product and to remove the bound ligands, the QIA quick nucleotide purification kit (Qiagen) was used according to the manufacturer’s instructions. This kit is specially designed for the purification of both double- and single-stranded ODNs from 17 bases in length. It employs a high-salt buffer to bind the negatively charged ODNs to the positively charged spin tube membrane through centrifugation so that all other components, including positively charged and neutral ligand molecules, are eluted. PCR-grade water was then used (rather than the manufacturer’s recommendation of an ethanol based buffer) to wash any impurities away before elution of the DNA using a low-salt concentration solution. The purified samples were freeze-dried and then re-dissolved in PCR-grade water at room temperature prior to the second amplification step.

#### Step 3

The purified extended samples were then subjected to PCR amplification. For this, a second PCR master mix was prepared consisting of 1 µM ACX reverse primer (5′-GCG CGG [CTTACC]_3_ CTA ACC-3′), 0.1 µg TS forward primer (5′-AAT CCG TCG AGC AGA GTT-3′), TRAP buffer, 5 µg BSA, 0.5 mM dNTPs, and 2 U *Taq* polymerase. A 6 µl aliquot of the master mix was added to the purified telomerase extended samples and amplified for 30 cycles of: 30 s at 94°C and 30 s at 59°C. The reaction products were loaded onto a 10% polyacrylamide gel (19∶1) in TBE 0.5 X. Gels were transferred to Whatman 3 mm paper, dried under vacuum at 80°C, and read using a phosphorimager 840 (Amersham). Measurements were made in triplicate with respect to a negative control run using the equivalent TRAP-PCR conditions but omitting the protein extract, thus ensuring that the ladders observed were not due to artefacts of the PCR reaction.

### Cell Viability Assay

HeLa (cervical cancer human transformed cells), A549 (lung carcinoma human transformed cells) and NIH3T3 (mouse embryonic fibroblast cells) cells were seeded in 96-well plates (15.0 × 10^3^/well). Cells were grown for 24 h before treatment to get >70% confluency and exposed to various concentrations of ligands in presence of 0.2% FBS. After either 6 h or 48 h incubation at 37°C in a humidified atmosphere of 5% CO_2_, old medium was replaced with the new one containing 10% FBS in DMEM and cells were further grown for 42 h post treatment. Then 20 µl of 5 mg/mL methyl thiazolyl tetrazolium (MTT) reagent was added to 200 µl of the medium present in each well and cells were further incubated for 4 h. Old medium was discarded and formazan crystals were dissolved in DMSO and reading (fluorescence) was taken at 595 nm in the ELISA plate reader. All ligand doses were parallel tested in triplicate. Percentage cell viability was calculated by using the formula,

% cell viability  =  [(FI_(595)_ of treated cells − FI_(595)_ of plain DMSO)/(FI_(595)_ of untreated cells − FI_(595)_ of plain DMSO)] ×100.

### Long-term Cell Culture Experiments

Long-term cell proliferation experiments were carried out using HeLa, A549 and NIH3T3 cell lines. Cells were grown in 6 well tissue culture plate at 5.0 × 10^4^ per well and exposed to a sub-cytotoxic concentration of (4 µM) or an equivalent volume of 0.1% DMSO every 5 days. The cells in control and ligand-exposed wells were counted and wells were reseeded with half population of the cells. This process was continued for 15 days. Cell population *vs.* time (days) plots were generated.

### Selectivity Toward Cancerous Cells

HEK293 (Human embryonic kidney 293) normal cells and HEK293T ((human embryonic kidney transformed 293) cancer cells were seeded in 96-well plates (8.0 × 10^3^/well). Experiments were performed as in the case of short term cell viability assay.

### Computational Methods

#### a) Preparation of dimeric G4DNA

The structures of different conformations of the basic G-quadruplex units (hybrid-1 and hybrid-2) were generated using the coordinates of the reported NMR structures (PDB code 2HY9 and 2JPZ) [10]. To make the 50-mer dimeric hybrid G4DNA structure, first we removed one adenine from 3′-end of the hybrid-1 structure (2HY9) and one thymine from the 5′-end of the hybrid-2 structure (2JPZ). Then we joined the 3′-end of the hybrid-1G- quadruplex unit with the 5′-end of the hybrid-2 G-quadruplex unit using *ChemCraft* software. In each G-quadruplex units, two K^+^ ions were placed between the adjacent G-tetrad planes. In this hybrid-type model, the monomeric G-quadruplex units were initially disposed to maximize the stacking interactions between the loop residues. Molecular dynamics simulations were performed using the ff99SB force field in the AMBER v9.0 package [57]. The models were solvated in a 10 Å box of TIP3P water using standard Amber 9.0 leap rules to hydrate the systems and K^+^ ions were added for overall charge neutrality [58]. The systems were heated slowly and equilibrated for 250 ps with gradual removal of the positional restraints on DNA following the protocol given below. We (i) minimized water and metal counter ions with DNA fixed; (ii) minimized total system; (iii) heated slowly from 0 to 300 K (100 kcal/mol Å^−1^) for 50 ps holding DNA fixed; we performed (iv) 50 ps MD (T = 300 K) holding DNA fixed (100 kcal/mol Å^−1^), (v) 50 ps MD (T = 300 K) holding DNA fixed (50 kcal/mol Å^−1^), (vi) 50 ps MD (T = 300 K) holding DNA fixed (10 kcal/mol Å^−1^) and (vii) 50 ps MD (T = 300 K) holding DNA fixed (1 kcal/mol Å^−1^). Subsequently, under constant pressure, production run of MD simulation of 8 ns was performed in an NPT ensemble at 1 atm and 300 K. The output and the trajectory files were saved every 2 fs for the subsequent analysis, respectively. All trajectory analysis was done with the Ptraj module in the Amber 9.0 suite and examined visually using the VMD software package [59].

#### b) Preparation of ligands and ligand-DNA complex

These were performed as described earlier [38].

#### c) Molecular modeling of the ligand-DNA complex

Partial-atomic charges for the ligand molecules were derived using the HF/6-31G* basis set followed by RESP calculation, while force-field parameters were taken from the generalized Amber force field (GAFF) using ANTECHAMBER module [60]. In each G-quadruplex units, two K^+^ ions were placed between the adjacent G-tetrad planes. The models were solvated in a 10 Å box of TIP3P water using standard Amber 9.0 leap rules to hydrate the systems and K^+^ ions were added for overall charge neutrality [57]. The systems were heated slowly and equilibrated for 250 ps with gradual removal of the positional restraints on DNA followed by the previous protocol (preparation of dimeric G4DNA).

## Supporting Information

Figure S1 **CD spectral titrations of the pre-formed G4DNA formed with ODN Hum_24_ and Hum_48_ in LiCl solution.** CD titrations of the pre-formed G4DNA derived from the Hum_48_ [(A) and (B): 4 µM strand concn.] or the Hum_24_ [(C): 4 µM strand concn.] in LiCl buffer (10 mM Tris-HCl, pH 7.4 having 100 mM LiCl) with indicated ligands and concentrations.
(TIF)

Figure S2 **CD spectral titrations of the pre-formed G4DNA formed with ODN Hum_24_ in (Na^+^ + LiCl) solution.** CD titrations of the pre-formed G4 DNA (2 µM strand conc.) formed with the Hum_24_ in (Na^+^ + LiCl) solution (10 mM sodium cacodylate having 100 mM LiCl, Panel A) or in KCl buffer (10 mM Tris-HCl, pH 7.4 having 100 mM KCl, Panel B) with **D3** at ligand:DNA ratio (r) = 5, 10, 15, 20, 25 respectively. Arrows indicate the increment in the CD intensity.
(TIF)

Figure S3 **CD spectral titrations of the pre-formed G4DNA formed with the Hum_48_ in (Na^+^ + LiCl) solution.** CD titrations of the pre-formed G4DNA (2 µM strand concn.) from the Hum_48_ in (Na^+^ + LiCl) solution (10 mM sodium cacodylate having 100 mM LiCl with **M** (Panel A) and **D3** (Panel B) at ligand: DNA ratio (r) = 5, 10, 15, 20, 25 for **M** and r = 5, 10, 15 for **D3** respectively. Arrows show the increment in the CD intensity.
(TIF)

Figure S4 **Representative CD melting profiles of the G4DNAs in (Na^+^ + LiCl) solution.** CD melting at 295 nm of the pre-formed G4DNA made with the A. Hum_24_ (4 µM) and B. Hum_48_ (2 µM) in (Na^+^ + LiCl) solution at 295 nm. G4DNA alone (Red) or complexed with the indicated ligand (10 equiv.). In case of **M**-Hum_48_, [**M]** was 20 equiv.
(TIF)

Figure S5 **Representative CD melting profiles of the G4DNAs in NaCl solution.** CD melting at 295 nm of the pre-formed G4DNA made with A. Hum_24_ (4 µM) and B. Hum_48_ (2 µM) in NaCl solution (10 mM Tris-HCl, pH 7.4 having 100 mM NaCl); G4DNA alone (Red) or complexed with each ligand (10 equiv.). In case of **M**-Hum_48_, [**M]** was 20 equiv.
(TIF)

Figure S6 **Representative CD melting profiles of the pre-formed G4DNAs in KCl solution.** CD melting at 295 nm of the pre-formed G4DNA made with the A. Hum_24_ (4 µM) and B. Hum_48_ (2 µM) in KCl solution (10 mM Tris-HCl, pH 7.4 having 100 mM KCl) at 295 nm; G4DNA alone (Red) or complexed with each ligand (10 equiv.). In case of **M**-Hum_48_, [**M**] was 20 equiv.
(TIF)

Figure S7 **CD melting profiles of G4DNA formed in presence of each ligand in KCl solution.** CD melting at 266 nm profiles of G4DNA made with the (A) Hum_24_ (4 µM) and (B) Hum_48_ (2 µM). G4DNA was either formed alone (Red) or formed in presence of the indicated ligand (10 equiv., first heated to 95°C for 5 min and then cooled slowly to room temperature) in KCl solution (10 mM Tris-HCl, pH 7.4 having 100 mM KCl). In case of **M**-Hum_48_, [**M]** was 20 equiv. Spectral changes were monitored at 295 nm for DNA alone and at 266 nm for the DNA-ligand complexes. (C) Cooling curves of the Hum_48_ DNA alone (red, at 295 nm) and with 10 equiv. of **D3** (wine, at 266 nm).
(TIF)

Figure S8 **UV-visible absorption titration of the gemini ligands with pre-formed G4DNAs.** UV-visible absorption spectra of three gemini ligands (A). UV-visible absorption titration of **D3** in presence of Hum48 (B) and CT-DNA.
(TIF)

Figure S9 **Scatchard plots to determine the binding affinity constant.** Scatchard binding plots (r/C_f_ *vs.* r) used to determine the affinity constants for: A) **D1**, B) **D2** and C) **D3** with the preformed Hum_48_ G4DNA in 100 mM KCl buffer.
(TIF)

Figure S10 **Fluorescence titrations of D3.** (A) Fluorescence titrations of **D3** alone with K^+^ solution in Tris-HCl buffer, pH 7.4 at 25°C (λ_ex_ = 380 nm). (B) Relative increments in the fluorescence intensity of each gemini ligand (500 nM) upon addition of the pre-formed Hum_48_ G4DNA in KCl buffer (10 mM Tris-HCl having 100 mM KCl and 0.1 mM EDTA, pH 7.4). (C) Representative emission spectra of titrations with **D3**. G4DNA formed by the Hum_48_ was added from a stock of 4 µM strand concentration to the solution of the ligand in the cuvette.
(TIF)

Figure S11 **Conventional two-step TRAP assay.** Telomerase inhibitory properties by various bisbenzimidazole ligands. ConTRAP assay was performed using indicated concentrations over each lane with **M** and **D1** (left panel); **D2** and **D3** (middle panel). Lane 1*:* (-) ve control, (absence of enzyme and ligand); Lane 2: R  =  PCR control and Lane 11: (+) ve control (absence of ligand) in both the cases. Other lanes (lanes 3-10) contain TRAP reaction mixtures mixed with indicated concentrations (µM) of each ligand. The rightmost panel shows the inhibition of taq polymerase by the ligand **D3** (Lanes 9 and 10) at 15 and 30 µM concentrations.
(TIF)

Figure S12 **Inhibition curves from TRAP-LIG assays for the complexes D1-3 and M.**
(TIF)

Figure S13 **Short term cell viability assay.** Effect of different ligands on the cell viability after short-term exposure (for 6 h and 48 h) of HeLa, A549 and NIH3T3 cells at specified concentrations as measured from the MTT assay. Each experiment was performed three times and an average at each point is shown.
(TIF)

Figure S14 **Long term cell viability assay.** Long-term exposure of HeLa, A549 and NIH3T3 cells with the indicated ligands at sub-cytotoxic concentrations. Cells were exposed to 4 µM of each ligand or 0.1% DMSO, respectively. Every 5 days, the cells in control and ligand-exposed wells were counted and wells reseeded with cells. Each experiment was performed three times at each point.
(TIF)

Figure S15 **Short term cell viability assay of 5, 10, 15, 20-tetrakis (1-methyl-4-pyridyl)-21H, 23H-porphine) (H2TMPyP4).** Effect of this ligand on the cell viability after short-term exposure (for 6 h and 48 h) of HeLa, A549 and NIH3T3 cells at specified concentrations as measured by MTT assay. Each experiment was performed three times and an average at each point is shown.
(TIF)

Figure S16 **In-silico construction of the dimeric G4DNA.** Dimeric G4DNA is depicted which was formed by joining two monomeric hybrid structure, hybrid-1 (2HY9) and hybrid-2 (2JPZ). Structure of dimeric G4DNA was constructed using *ChemCraft* software.
(TIF)

Figure S17 **Simulated structure of dimeric G4DNA.** Simulated structure of dimeric G4DNA (8 ns). K^+^ ions present in the central cavity are shown by cyan colour. Hybrid G4-DNA is represented as a cartoon form (green-orange).
(TIF)

Figure S18 **RMSD values of the ligands.** RMSD plots of dimeric G4DNA and ligand-G4DNA complexes.
(TIF)

Figure S19 **Effect of ligands on the cell viability toward normal and transformed cells at one specific ligand concentration.** Selective % cell viability of normal (HEK293) against transformed (HEK293T) cells by monomer (**M**) and the gemini ligands (**D1**, **D2** and **D3**) at a particular concentration (30 µM).
(TIF)

Table S1 **Melting temperatures (at 257 nm) of the Hum_24_ and Hum_48_ G4DNAs formed in LiCl solution and G4DNA-ligand complexes ([ligand]: [DNA] ratio ‘r’  =  10 for M and 5 for the gemini ligands).**
(DOCX)

Table S2 **Dissociation constants (K_D_) of the gemini ligands with the pre-formed Hum_48_ G4DNA and CT-DNA determined from UV-vis absorption spectral titrations.**
(DOC)

Table S3 **IC_50_ values (µM, ±5%) of the ligands against different cell lines after 48 h treatment.**
(DOC)

Text S1 **Synthesis and characterization of the ligands used in this study.**
(DOC)
