# Supplementary figures and images for: Binding of Gemini Bisbenzimidazole Drugs with Human Telomeric G-Quadruplex Dimers: Effect of the Spacer in the Design of Potent Telomerase Inhibitors
Source: PLoS One. 2012 Jun 21;7(6):e39467. doi: 10.1371/journal.pone.0039467 (PMC3380826; doi:10.1371/journal.pone.0039467)

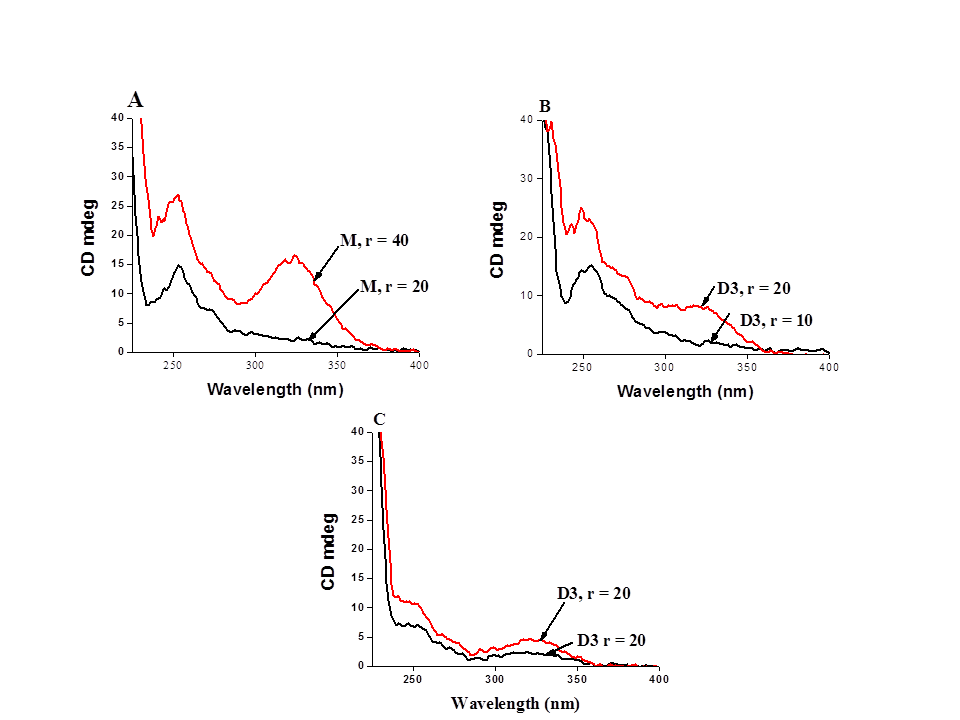

Supplement: Figure S1 — CD spectral titrations of the pre-formed G4DNA formed with ODN Hum24 and Hum48 in LiCl solution. CD titrations of the pre-formed G4DNA derived from the Hum48 [(A) and (B): 4 µM strand concn.] or the Hum24 [(C): 4 µM strand concn.] in LiCl buffer (10 mM Tris-HCl, pH 7.4 having 100 mM LiCl) with indicated ligands and concentrations. (TIF) [file pone.0039467.s001.tif]

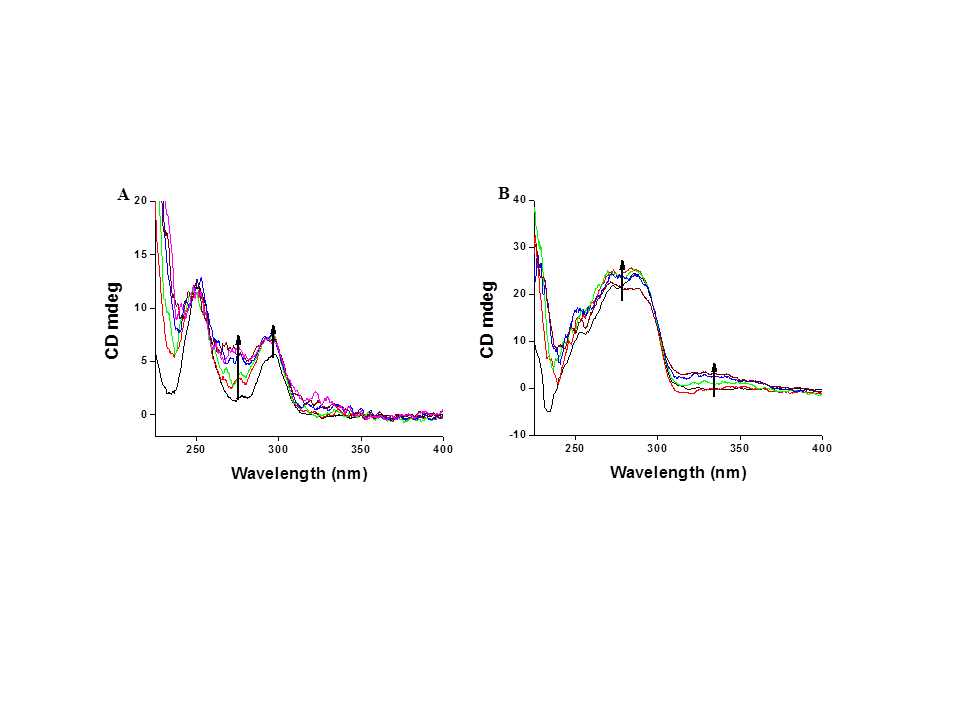

Supplement: Figure S2 — CD spectral titrations of the pre-formed G4DNA formed with ODN Hum24 in (Na+ + LiCl) solution. CD titrations of the pre-formed G4 DNA (2 µM strand conc.) formed with the Hum24 in (Na+ + LiCl) solution (10 mM sodium cacodylate having 100 mM LiCl, Panel A) or in KCl buffer (10 mM Tris-HCl, pH 7.4 having 100 mM KCl, Panel B) with D3 at ligand:DNA ratio (r) = 5, 10, 15, 20, 25 respectively. Arrows indicate the increment in the CD intensity. (TIF) [file pone.0039467.s002.tif]

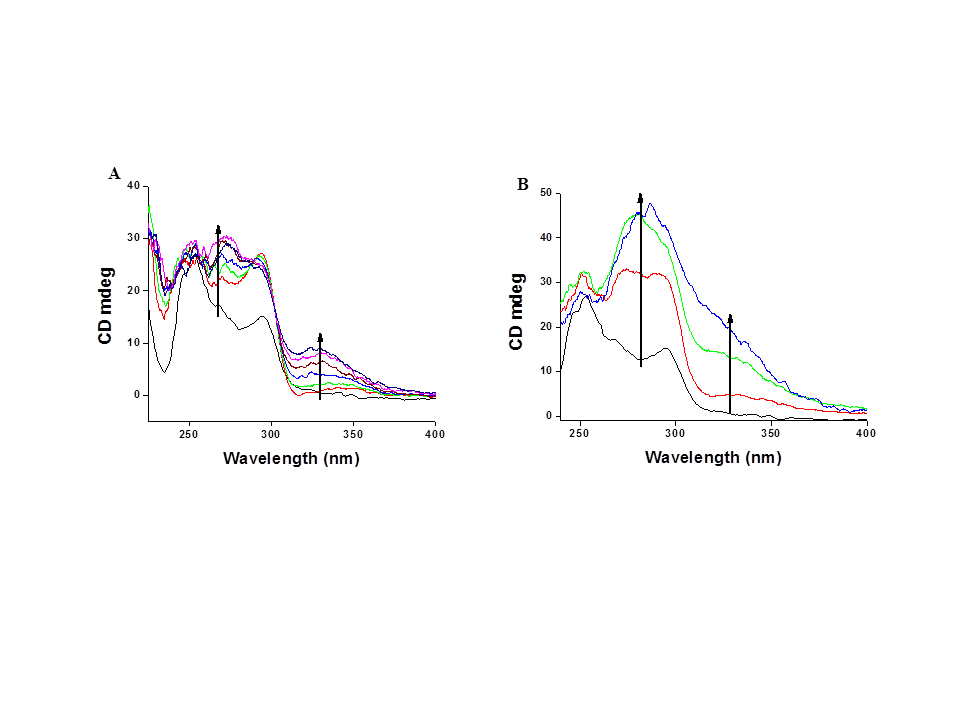

Supplement: Figure S3 — CD spectral titrations of the pre-formed G4DNA formed with the Hum48 in (Na+ + LiCl) solution. CD titrations of the pre-formed G4DNA (2 µM strand concn.) from the Hum48 in (Na+ + LiCl) solution (10 mM sodium cacodylate having 100 mM LiCl with M (Panel A) and D3 (Panel B) at ligand: DNA ratio (r) = 5, 10, 15, 20, 25 for M and r = 5, 10, 15 for D3 respectively. Arrows show the increment in the CD intensity. (TIF) [file pone.0039467.s003.tif]

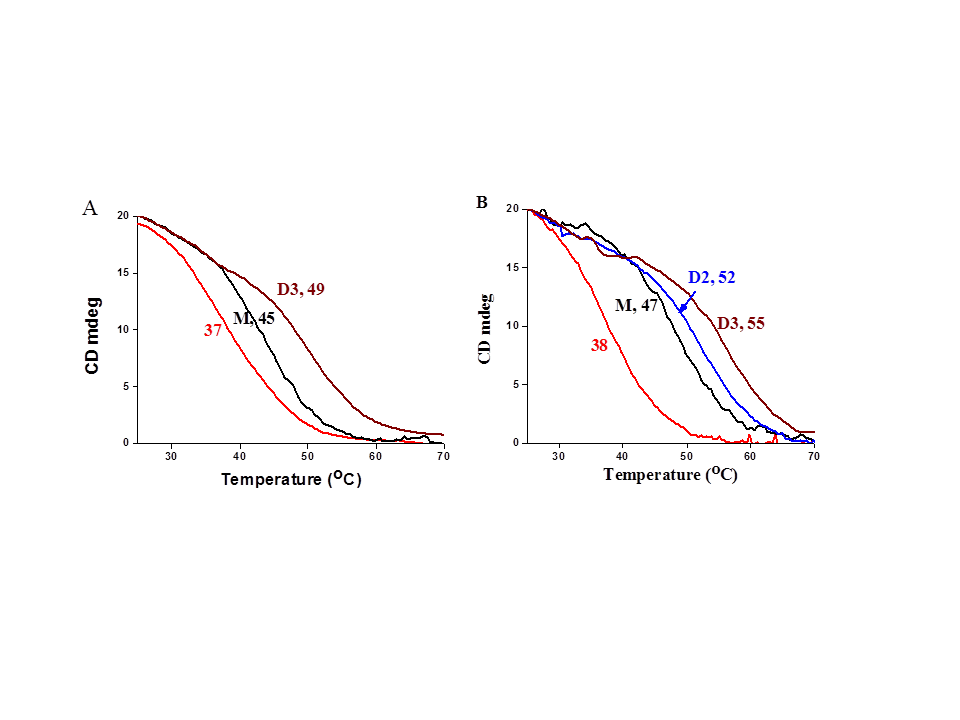

Supplement: Figure S4 — Representative CD melting profiles of the G4DNAs in (Na+ + LiCl) solution. CD melting at 295 nm of the pre-formed G4DNA made with the A. Hum24 (4 µM) and B. Hum48 (2 µM) in (Na+ + LiCl) solution at 295 nm. G4DNA alone (Red) or complexed with the indicated ligand (10 equiv.). In case of M-Hum48, [M] was 20 equiv. (TIF) [file pone.0039467.s004.tif]

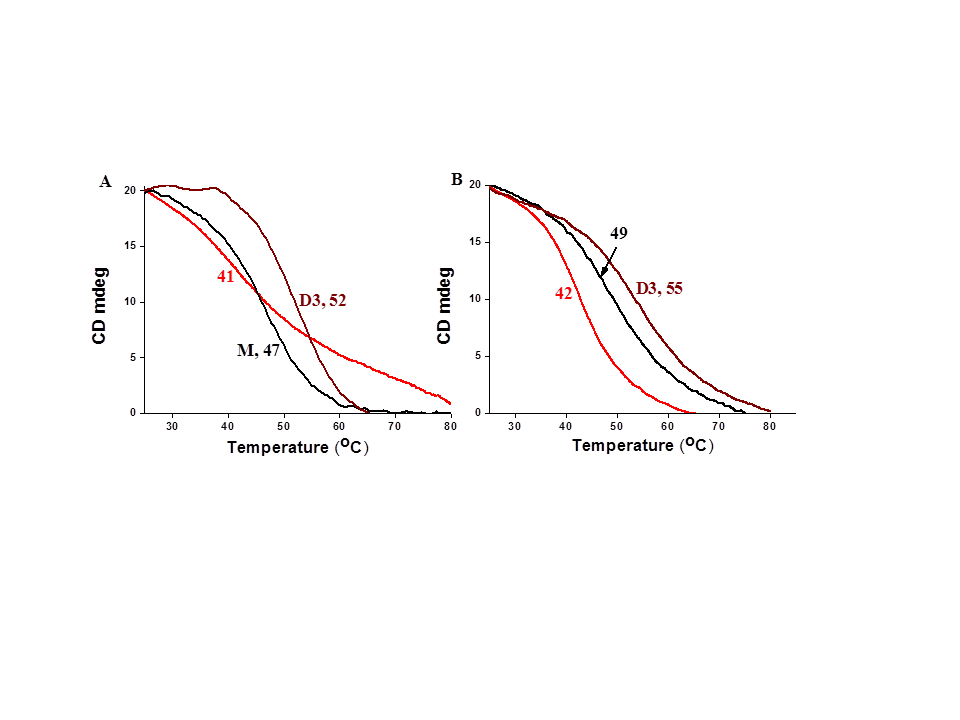

Supplement: Figure S5 — Representative CD melting profiles of the G4DNAs in NaCl solution. CD melting at 295 nm of the pre-formed G4DNA made with A. Hum24 (4 µM) and B. Hum48 (2 µM) in NaCl solution (10 mM Tris-HCl, pH 7.4 having 100 mM NaCl); G4DNA alone (Red) or complexed with each ligand (10 equiv.). In case of M-Hum48, [M] was 20 equiv. (TIF) [file pone.0039467.s005.tif]

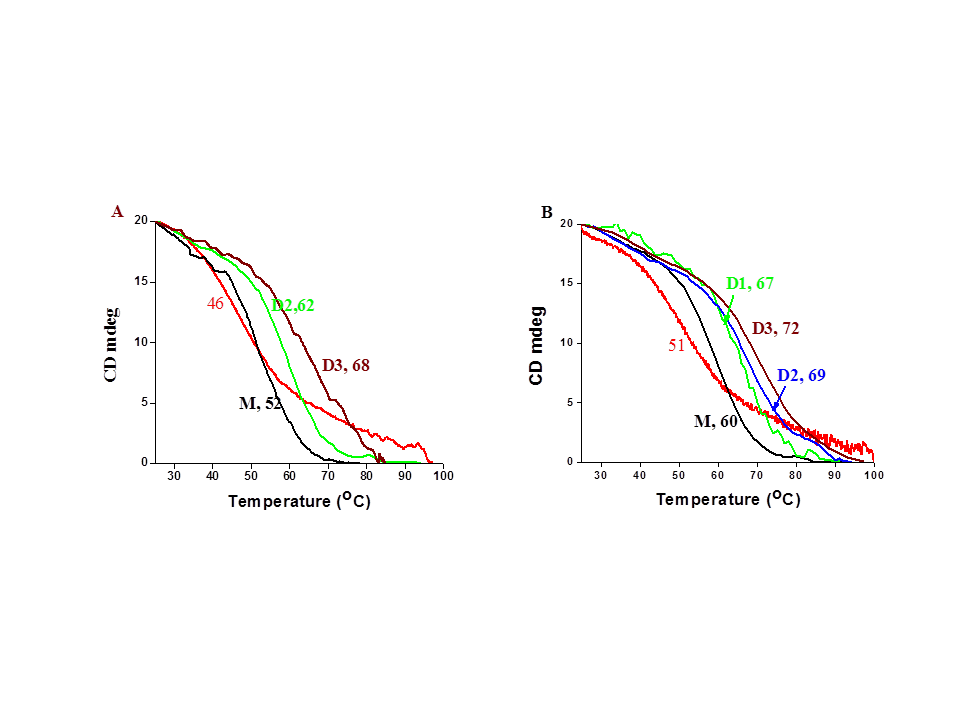

Supplement: Figure S6 — Representative CD melting profiles of the pre-formed G4DNAs in KCl solution. CD melting at 295 nm of the pre-formed G4DNA made with the A. Hum24 (4 µM) and B. Hum48 (2 µM) in KCl solution (10 mM Tris-HCl, pH 7.4 having 100 mM KCl) at 295 nm; G4DNA alone (Red) or complexed with each ligand (10 equiv.). In case of M-Hum48, [M] was 20 equiv. (TIF) [file pone.0039467.s006.tif]

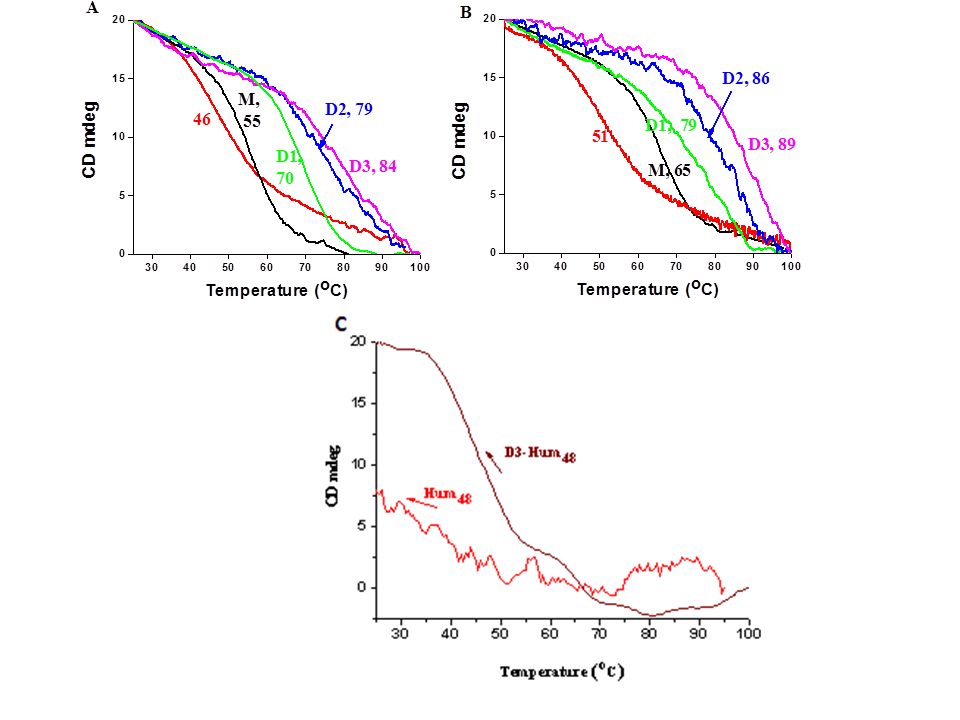

Supplement: Figure S7 — CD melting profiles of G4DNA formed in presence of each ligand in KCl solution. CD melting at 266 nm profiles of G4DNA made with the (A) Hum24 (4 µM) and (B) Hum48 (2 µM). G4DNA was either formed alone (Red) or formed in presence of the indicated ligand (10 equiv., first heated to 95°C for 5 min and then cooled slowly to room temperature) in KCl solution (10 mM Tris-HCl, pH 7.4 having 100 mM KCl). In case of M-Hum48, [M] was 20 equiv. Spectral changes were monitored at 295 nm for DNA alone and at 266 nm for the DNA-ligand complexes. (C) Cooling curves of the Hum48 DNA alone (red, at 295 nm) and with 10 equiv. of D3 (wine, at 266 nm). (TIF) [file pone.0039467.s007.tif]

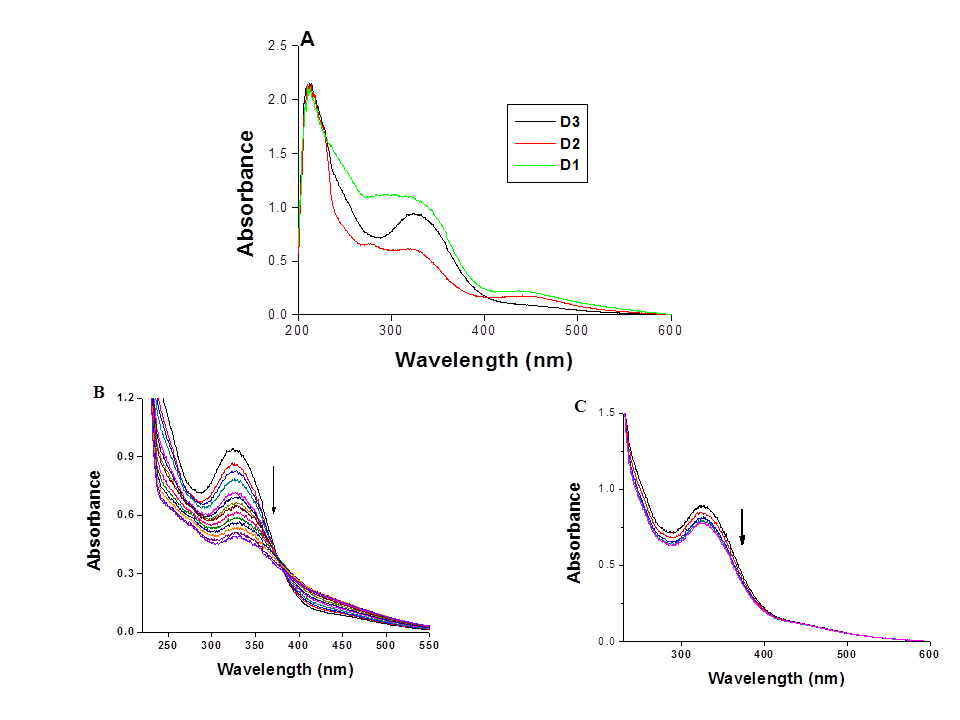

Supplement: Figure S8 — UV-visible absorption titration of the gemini ligands with pre-formed G4DNAs. UV-visible absorption spectra of three gemini ligands (A). UV-visible absorption titration of D3 in presence of Hum48 (B) and CT-DNA. (TIF) [file pone.0039467.s008.tif]

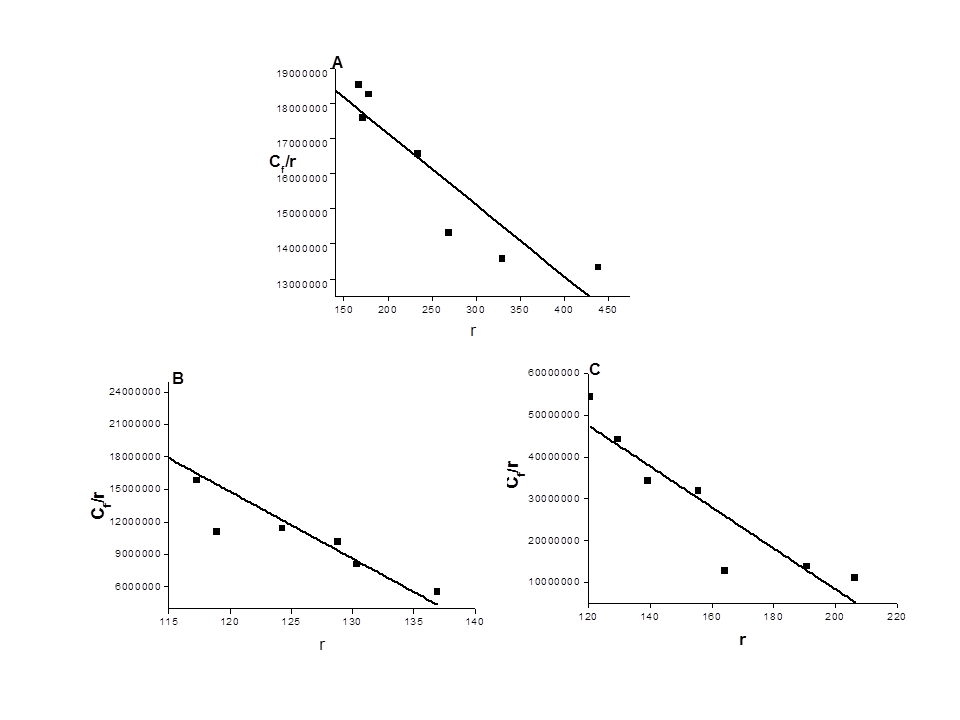

Supplement: Figure S9 — Scatchard plots to determine the binding affinity constant. Scatchard binding plots (r/Cf vs. r) used to determine the affinity constants for: A) D1, B) D2 and C) D3 with the preformed Hum48 G4DNA in 100 mM KCl buffer. (TIF) [file pone.0039467.s009.tif]

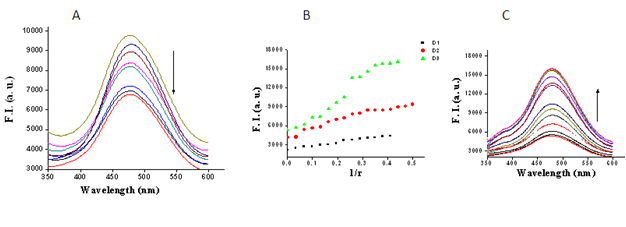

Supplement: Figure S10 — Fluorescence titrations of D3. (A) Fluorescence titrations of D3 alone with K+ solution in Tris-HCl buffer, pH 7.4 at 25°C (λex = 380 nm). (B) Relative increments in the fluorescence intensity of each gemini ligand (500 nM) upon addition of the pre-formed Hum48 G4DNA in KCl buffer (10 mM Tris-HCl having 100 mM KCl and 0.1 mM EDTA, pH 7.4). (C) Representative emission spectra of titrations with D3. G4DNA formed by the Hum48 was added from a stock of 4 µM strand concentration to the solution of the ligand in the cuvette. (TIF) [file pone.0039467.s010.tif]

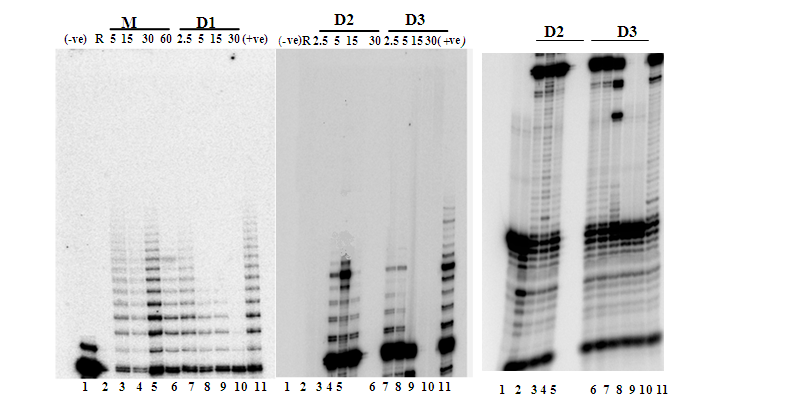

Supplement: Figure S11 — Conventional two-step TRAP assay. Telomerase inhibitory properties by various bisbenzimidazole ligands. ConTRAP assay was performed using indicated concentrations over each lane with M and D1 (left panel); D2 and D3 (middle panel). Lane 1: (-) ve control, (absence of enzyme and ligand); Lane 2: R = PCR control and Lane 11: (+) ve control (absence of ligand) in both the cases. Other lanes (lanes 3-10) contain TRAP reaction mixtures mixed with indicated concentrations (µM) of each ligand. The rightmost panel shows the inhibition of taq polymerase by the ligand D3 (Lanes 9 and 10) at 15 and 30 µM concentrations. (TIF) [file pone.0039467.s011.tif]

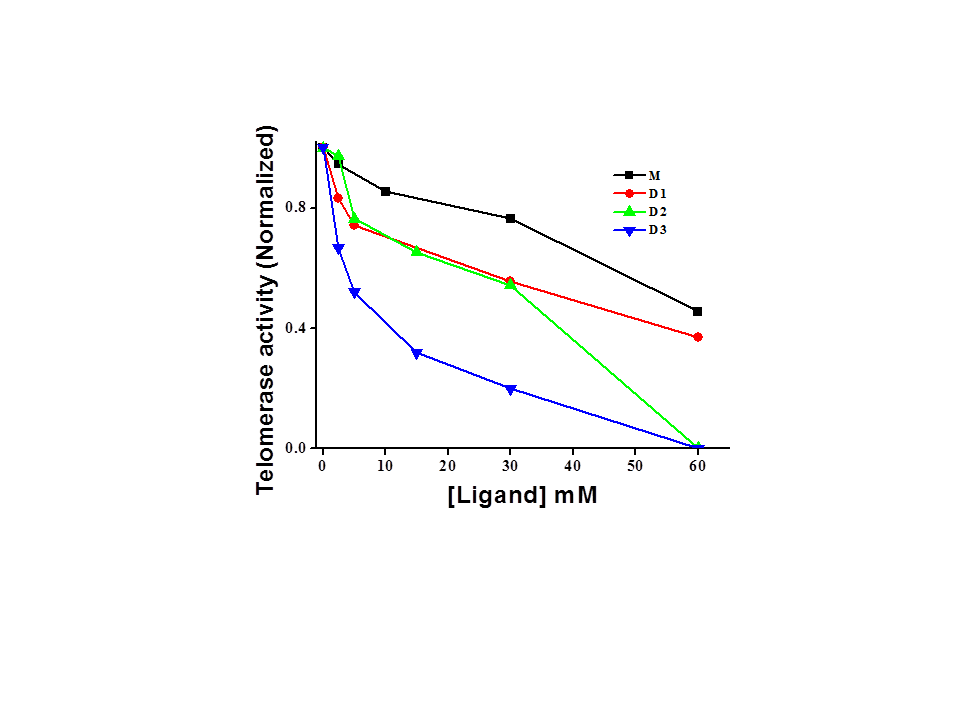

Supplement: Figure S12 — Inhibition curves from TRAP-LIG assays for the complexes D1-3 and M. (TIF) [file pone.0039467.s012.tif]

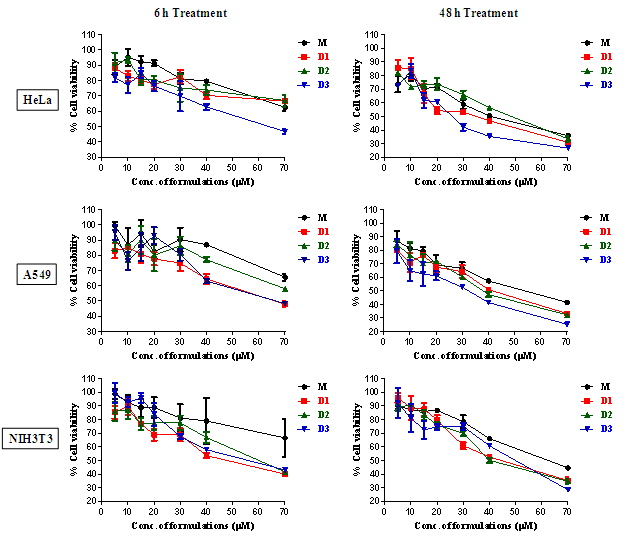

Supplement: Figure S13 — Short term cell viability assay. Effect of different ligands on the cell viability after short-term exposure (for 6 h and 48 h) of HeLa, A549 and NIH3T3 cells at specified concentrations as measured from the MTT assay. Each experiment was performed three times and an average at each point is shown. (TIF) [file pone.0039467.s013.tif]

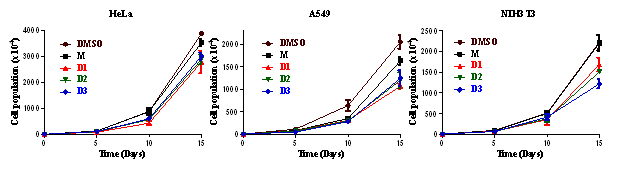

Supplement: Figure S14 — Long term cell viability assay. Long-term exposure of HeLa, A549 and NIH3T3 cells with the indicated ligands at sub-cytotoxic concentrations. Cells were exposed to 4 µM of each ligand or 0.1% DMSO, respectively. Every 5 days, the cells in control and ligand-exposed wells were counted and wells reseeded with cells. Each experiment was performed three times at each point. (TIF) [file pone.0039467.s014.tif]

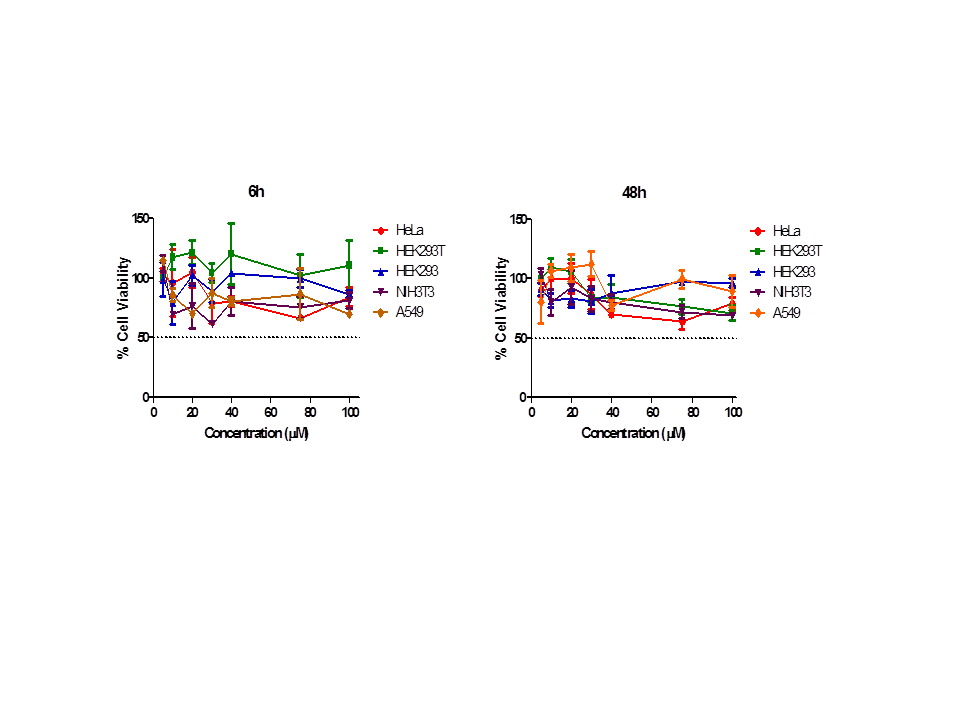

Supplement: Figure S15 — Short term cell viability assay of 5, 10, 15, 20-tetrakis (1-methyl-4-pyridyl)-21H, 23H-porphine) (H2TMPyP4). Effect of this ligand on the cell viability after short-term exposure (for 6 h and 48 h) of HeLa, A549 and NIH3T3 cells at specified concentrations as measured by MTT assay. Each experiment was performed three times and an average at each point is shown. (TIF) [file pone.0039467.s015.tif]

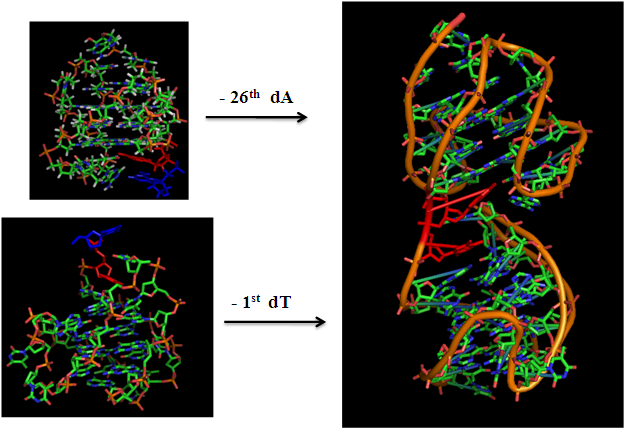

Supplement: Figure S16 — In-silico construction of the dimeric G4DNA. Dimeric G4DNA is depicted which was formed by joining two monomeric hybrid structure, hybrid-1 (2HY9) and hybrid-2 (2JPZ). Structure of dimeric G4DNA was constructed using ChemCraft software. (TIF) [file pone.0039467.s016.tif]

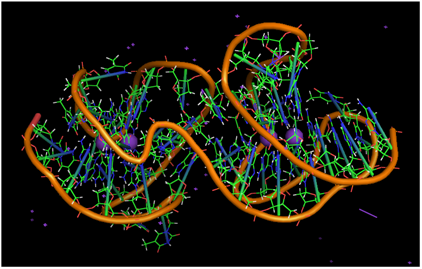

Supplement: Figure S17 — Simulated structure of dimeric G4DNA. Simulated structure of dimeric G4DNA (8 ns). K+ ions present in the central cavity are shown by cyan colour. Hybrid G4-DNA is represented as a cartoon form (green-orange). (TIF) [file pone.0039467.s017.tif]

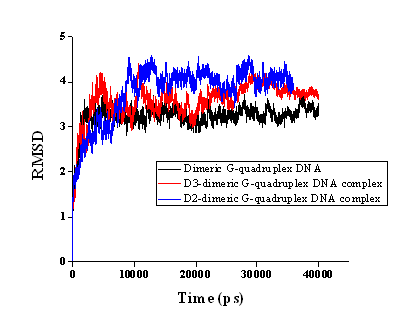

Supplement: Figure S18 — RMSD values of the ligands. RMSD plots of dimeric G4DNA and ligand-G4DNA complexes. (TIF) [file pone.0039467.s018.tif]

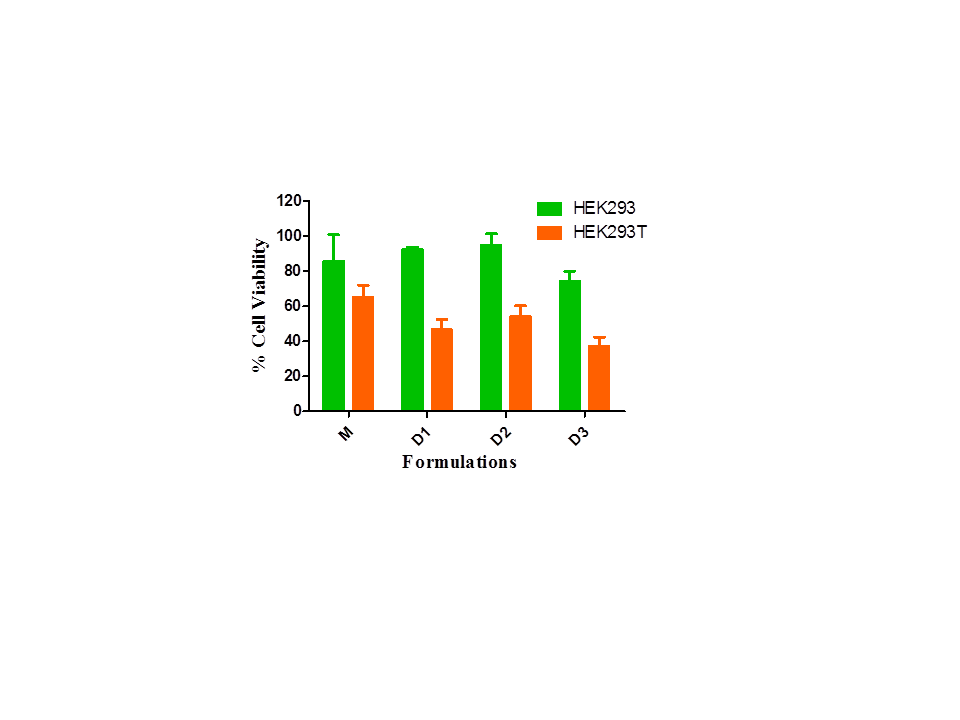

Supplement: Figure S19 — Effect of ligands on the cell viability toward normal and transformed cells at one specific ligand concentration. Selective % cell viability of normal (HEK293) against transformed (HEK293T) cells by monomer (M) and the gemini ligands (D1, D2 and D3) at a particular concentration (30 µM). (TIF) [file pone.0039467.s019.tif]
