# Supplementary material for: Binding of Gemini Bisbenzimidazole Drugs with Human Telomeric G-Quadruplex Dimers: Effect of the Spacer in the Design of Potent Telomerase Inhibitors
Source: PLoS One. 2012 Jun 21;7(6):e39467. doi: 10.1371/journal.pone.0039467 (PMC3380826; doi:10.1371/journal.pone.0039467)
Supplement: Table S1 — Melting temperatures (at 257 nm) of the Hum24 and Hum48 G4DNAs formed in LiCl solution and G4DNA-ligand complexes ([ligand]: [DNA] ratio ‘r’ = 10 for M and 5 for the gemini ligands). (DOCX) [file pone.0039467.s020.docx]

**Table S1. Melting temperatures^a^ of Hum_24_ and Hum_48_ G4DNAs formed in 10 mM Tris-HCl, pH 7.4 buffer having 100 mM LiCl and 0.1 mM EDTA. The G4DNA-ligand complexes have a [ligand]:[DNA] ratio ‘r’ = 10.**

| **Entry** | **ODN/ODN-ligand** | **LiCl solution** | |
| --- | --- | --- | --- |
|  |  | ***T*_m_^o^C** | **Δ*T*_m_ ^o^C** |
| 1 | Hum_24_ | 36 | - |
| 2 | Hum_24_ + **D3** | 41 | 5 |
| 3 | Hum_48_ | 37 | - |
| 4 | Hum_48_ + **D3** | 47 | 10 |

^_____________________________________________________________________________________________________________________^

**^a^**Changes in the circular dichroism spectral peak at 295 nm monitored as a function of temperature using 2 µM strand ODN concentration. The results are average of two experiments and are within ± 0.5 ^o^C of each other.
