# Supplementary material for: Binding of Gemini Bisbenzimidazole Drugs with Human Telomeric G-Quadruplex Dimers: Effect of the Spacer in the Design of Potent Telomerase Inhibitors
Source: PLoS One. 2012 Jun 21;7(6):e39467. doi: 10.1371/journal.pone.0039467 (PMC3380826; doi:10.1371/journal.pone.0039467)
Supplement: Table S2 — Dissociation constants (KD) of the gemini ligands with the pre-formed Hum48 G4DNA and CT-DNA determined from UV-vis absorption spectral titrations. (DOC) [file pone.0039467.s021.doc]

**Table S2.** Dissociation constants (K_D_) of the complexes of the gemini ligands with the pre-formed Hum_48_ G4DNA^a^ and CT DNA^b^

Ligand K_D_ (10^5^ M^-1^)

Hum_48_ Duplex

**D1** 0.82 ± 0.3 0.09 ± 0.4

**D2** 4.92 ± 0.2 0.06 ± 0.1

**D3** 6.16 ± 0.2 0.08 ± 0.2

*^a^*Binding assays were performed with pre-formed Hum_48_ G4DNA in 10 mM Tris-HCl, having 100 mM KCl and 0.1 mM EDTA buffer at pH7.3 and with *^b^*CT-DNA in 10 mM Tris-HCl, having 100 mM NaCl and 0.1 mM EDTA buffer at pH 7.3.
