# Supplementary material for: Binding of Gemini Bisbenzimidazole Drugs with Human Telomeric G-Quadruplex Dimers: Effect of the Spacer in the Design of Potent Telomerase Inhibitors
Source: PLoS One. 2012 Jun 21;7(6):e39467. doi: 10.1371/journal.pone.0039467 (PMC3380826; doi:10.1371/journal.pone.0039467)
Supplement: Table S3 — IC50 values (µM, ±5%) of the ligands against different cell lines after 48 h treatment. (DOC) [file pone.0039467.s022.doc]

**Table S3.** IC_50_ values (µM) of various ligands against different cell lines after 48 h treatment.^a^

**Cells**  **IC_50_ (µM)**

**M D1 D2 D3**

HEK293 >150 >150 >150 >150

HEK293T 30 30 30 15

HeLa 40 35 45 22

A549 55 40 40 35

NIH3T3 65 40 42 45

^a^These values were obtained after performing each experiment in triplicate and are within ± 5%.
