# Supplementary material for: Binding of Gemini Bisbenzimidazole Drugs with Human Telomeric G-Quadruplex Dimers: Effect of the Spacer in the Design of Potent Telomerase Inhibitors
Source: PLoS One. 2012 Jun 21;7(6):e39467. doi: 10.1371/journal.pone.0039467 (PMC3380826; doi:10.1371/journal.pone.0039467)
Supplement: Text S1 — Synthesis and characterization of the ligands used in this study. (DOC) [file pone.0039467.s023.doc]

**Experimental Procedures.**

**General Spectrometric Characterizations.** ^1^H and ^13^C NMR spectra were recorded on Bruker AMX (300 MHz or 400 MHz) spectrometers. IR spectra were recorded on FT-IR Perkin Elmer GX spectrometer. Melting points were taken on open capillaries inside a Buchi melting point B540 apparatus and are uncorrected. Mass spectra were recorded on Micromass Q-TOF Micro TM spectrometer. MALDI mass spectra were recorded on ultra flex TOF/TOF mass spectrometer (Bruker Daltonics, Bremen, Germany) in the positive ion mode, using α-cyano-4-hydroxycinnamic acid as a matrix.

**Synthesis.** Monomeric compound **M** has been synthesized as reported previously.^1^ Dimeric compound (**D3**) has been synthesized as described below (**Schemes 1 and 2**).

**Hexaethyleneglycol ditosylate (2).** Hexaethylene glycol (**1**; 10 mmol) was dissolved in THF (10 mL) and NaOH (1.0 g, 25 mmol), dissolved in 2.5 mL of water was added to this under ice-cold conditions. The resulting solution was stirred for 30 min and then to this, *p*-tosyl chloride (4.7 g, 25 mmol), dissolved in 5 mL of THF was added dropwise. The mixture was allowed to come at room temperature and stirred for further 3h. Solvent was evaporated and water (20 mL) was added and extraction was done with ethyl acetate to get respective crude products. The crude products were then purified by column chromatography (EtOAc/Hexanes) on silica gel (60-120 mesh size) to obtain the required product (5.01 g, 85% yield). This was isolated as a colorless oil. IR: 2911, 2872, 1553, 1325, 1226, 1116, cm^-1^. ^1^H-NMR (CDCl_3_): δ 7.8 (d, 4H; *J* = 8 Hz), 7.36 (d, 4H; *J* = 8 Hz), 4.15 (t, 4H; *J* = 4.6 Hz), 3.69 (t, 4H; *J* = 5.0 Hz), 3.6 (bs, 16H), 2.45 (s, 6H); m/z (Q-TOF HRMS) found, 613.1760 (calculated, 613.1753, [M + Na]^+^). The NMR values were in agreement with the reported values.^2^

**1, 17-bis-[phenoxy (3, 5-dicarboxylic acid dimethyl ester)] 3, 6, 9, 12, 15-pentaoxo-hepta-decane (3).** Compound **2** (2.95 g, 5 mmol), 5-hydroxy-isophthalic acid dimethyl ester (2.3 g, 11 mmol) and K_2_CO_3_ (1.5 g) were refluxed in CH_3_CN (20 mL) with stirring for 10h. Reaction mixture was cooled, filtered and then evaporated. Residue was dissolved in water (50 mL) and extracted with ethyl acetate to furnish 3.1 g of the crude product. This crude material was then purified by column chromatography (EtOAc/Hexanes) on silica gel (60-120 mesh size) to obtain the required product (2.66 g, 80% yield) as colorless oil. IR: 2912.5, 2874, 1697, 1551, 1243, 1226.5 cm^-1^. ^1^H-NMR (CDCl_3_): δ 8.27 (s, 2H), 7.76 (d, 4H; *J* = 2 Hz), 4.21 (t, 4H; *J* = 4.5 Hz), 3.93 (s, 20H), 3.88 (t, 4H; *J* = 2 Hz), 3.66 (bs, 8H); m/z (Q-TOF HRMS) found, 689.2426 (calculated, 689.2421, [M + Na]^+^).

**1, 17-bis-[phenoxy (3, 5-dihydroxymethyl)] 3, 6, 9, 12, 15-pentaoxo-heptadecane (4)**. Compound **3** (999 mg, 1.5 mmol) was dissolved in THF (10 mL) and lithium aluminum hydride (171 mg, 9 mmol) was added under ice-cold conditions. Reaction mixture was allowed to come to room temperature and stirred for 12 h. Dilute HCl solution was added to decompose the un-reacted excess of LAH, stirred for half an hour and then resulting solution was extracted with ethyl acetate to give 573 mg of compound **4** as a off-white gum (69% yield). IR: 3413, 2956, 2926, 2242, 1642, 1456, 1293 cm^-1^. ^1^H-NMR (CDCl_3_): δ 6.76 (s, 2H), 6.72 (s, 4H), 4.48 (s, 8H), 4.02 (t, 4H; *J* = 4.5 Hz), 3.74 (t. 4H; *J* = 4.2 Hz), 3.57 (bs, 16H); m/z (Q-TOF HRMS) found, 577.2621 (calculated, 577.2625, [M + Na]^+^).

**1, 17-bis-[phenoxy (3, 5-dicarbaldehyde)] 3, 6, 9, 12, 15-pentaoxo-heptadecane (5)**. Compound **4** (277 mg, 0.5 m mol) was dissolved in a mixture of dichloromethane (DCM) and THF (6:4, 15 mL) and 6 equiv. of pyridinium chlorochromate (PCC) was added to this solution and some silica was also added. The mixture was stirred at room temperature for 1h and then directly loaded on to a silica gel column and the required product (194 mg, 71% yield) was eluted with DCM as yellowish oil. IR: 2924, 2872.5, 1695, 1596, 1464, 1293 cm^-1^. ^1^H-NMR (CDCl_3_): δ 10.0 (s, 2H), 7.96 (s, 2H), 7.67 (s, 4H), 4.24 (bs, 4H), 3.95 (bs, 4H), 3.71 (bs, 16H); m/z (Q-TOF HRMS) found, 569.1992 (calculated, 569.1999, [M + Na]^+^).

**1, 17-Bis [2, 2’-(5-phenoxy-1, 3-phenylene)-bis-[5-(4-(2-hydroxyethyl)-1-piperazinyl)-1H-benzimidazole]]-3, 6, 9, 12, 15-pentaoxo-heptadecane (D3).** To a freshly prepared solution of 4-[4-(2-hydroxyethyl)piperazin-1-yl]-benzene-1, 2-diamine (**6**, 188 mg, 0.8 m mol)^2^ in ethanol, 1/4 equivalent of the tetra aldehyde **5** (109 mg, 0.2 mmol) was added and to this solution, sodium metabisulphite (Na_2_S_2_O_5_, two equiv., 75 mg, dissolved in minimum quantity of water) was added. The reaction mixture was refluxed for 7-8 h with stirring, then cooled to room temperature, and filtered through celite. Solvent from the reaction mixture was then removed under reduced pressure to obtain the required crude product. This crude material was then purified by column chromatography (EtOAc/MeOH) on silica gel (70-220 mesh size) to obtain the required product. Isolated yield: 180 mg, 64%; mp: > 290 ^o^C; IR: 3411, 3017, 2870, 1618, 1565.5, 1452, 1222 cm^-1^; ^1^H-NMR δ 13.16 (b, 4H), 7.89 (bs, 4H), 7.50 (bs, 6H), 7.0 (bs, 8H), 5.39 (bs, 4H), 4.1 (bs, 12H), 3.9 (bs, 24H), 3.6 (bs, 24H), 3.25 (bs, 16 H); ^13^C NMR (DMSO-d6) δ: 159.22, 158.79, 150.2, 146.58, 132.15, 119.55, 118.91, 116.94, 114.29, 112.98, 79.24, 69.87, 68.89, 67.7, 57.89, 55.4, 51.53, 47.11, 42.0; m/z (MALDI-TOF) found, 1411.850 (calcd, 1411.853, [M + H]^+^); elemental (calcd. for C_76_H_98_N_16_O_11_.2H_2_O): C, 63.05; H, 7.10; N, 15.48; found: C, 63.0; H, 7.15; N, 15.39.

**Note:** Dimeric ligands **D1** and **D2** were also synthesized using similar synthetic protocol. Here we give their characterization data.^3^

**1, 11-Bis-[2, 2’-(5-phenoxy-1, 3-phenylene)-bis-[5-(4-(2-hydroxyethyl)-1-piperazinyl)-1H-benzimidazole]]-3, 6, 9-trioxo-undecane (D1).** Isolated yield: 163 mg, 62%; mp: >290 ^o^C; IR: 3410, 3016, 2878, 1614, 1565, 1450, 1220 cm^-1^; ^1^H-NMR δ 13.1 (b, 4H), 7.85 (bs, 4H), 7.5 (bs, 6H), 7.0 (bs, 8H), 4.3 (bs, 4H), 3.8 (bs, 12H), 3.68 (bs, 16H), 3.58 (bs, 24H), 3.2 (bs, 16 H); ^13^C NMR (DMSO-d6) δ: 159.19, 158.79, 150.24, 146.50, 132.09, 119.53, 116.95, 115.50, 114.24, 113.01, 79.24, 69.83, 68.86, 67.72, 57.88, 55.4, 51.51, 47.06, 42.0; m/z (Maldi-TOF) found, 1323.975 (calcd, 1323.978, [M + H]^+^); elemental (calcd. for C_72_H_90_N_16_O_9_.1.5H_2_O): C, 64.03; H, 6.94; N, 16.59; found: C, 63.98; H, 7.0; N, 16.50.

**1, 14-Bis-[2, 2’-(5-phenoxy-1, 3-phenylene)-bis-[5-(4-(2-hydroxyethyl)-1-piperazinyl)-1H-benzimidazole]]-3, 6, 9, 12-tetraoxo-tetradecane (D2).** Isolated yield: 164 mg, 60%; mp: > 290 ^o^C; IR: 3408, 3018, 2875, 1615, 1563, 1452, 1220 cm^-1^; ^1^H-NMR δ 13.12 (b, 4H), 7.89 (bs, 4H), 7.54 (bs, 6H), 7.0 (bs, 8H), 5.3 (bs, 4H), 4.0 (bs, 12H), 3.8 (bs, 20H), 3.58 (bs, 24H), 3.2 (bs, 16 H); ^13^C NMR (DMSO-d6) δ: 159.22, 158.79, 150.24, 146.59, 132.14, 119.76, 119.52, 116.91, 114.21, 112.97, 79.21, 69.87, 68.86, 67.69, 57.88, 55.4, 51.52, 47.1, 42.0; m/z (Maldi-TOF) found, 1367.962 (calcd, 1367.968, [M + H]^+^); elemental (calcd. for C_74_H_94_N_16_O_10_.2H_2_O): C, 63.32; H, 7.04; N, 15.97; found: C, 63.30; H, 7.1; N, 15.92.

**References.**

1. Jain AK, Reddy VV, Paul A, Muniyappa K, Bhattacharya S (2009) The synthesis and evolution of 1, 3-phenylene-bis(piperazinyl benzimidazole) derivatives: A novel class of G-quadruplex stabilizing small molecules in presence and absence of stabilizing cations Biochemistry 48: 10693-10704.
2. Lee C-S, Teng P-F, Wong W-L, Kwong H-L, Chan ASC (2005) New C2-symmetric 2, 20-bipyridine crown macrocycles for enantioselective recognition of amino acid derivatives. Tetrahedron 61: 7924–7930.
3. Jain AK, Paul A, Maji B, Muniyappa K, Bhattacharya S (2012) Dimeric 1,3-Phenylene-bis(piperazinyl benzimidazole)s: Synthesis and structure−activity investigations on their binding with human telomeric G-Quadruplex DNA and telomerase inhibition properties. J Med Chem 55: 2981−2993.

**Scheme 1.^a^**

**^a^Scheme1.** i) *p*-TsCl, NaOH, THF/H_2_O; ii) K_2_CO_3_, CH_3_CN, reflux; iii) LAH, THF, rt; iv) PCC, DCM or

DCM/THF, rt.

**Scheme 2.**

Na_2_S_2_O_5_, EtOH, reflux

**5**

+

**D3**

**6** R = CH_2_CH_2_OH

**D3** n = 5, R = CH_2_CH_2_OH
